# Perioperative stroke deteriorates white matter integrity by enhancing cytotoxic CD8 ^+^ T‐cell activation

**DOI:** 10.1111/cns.14747

**Published:** 2024-07-07

**Authors:** Yuxi Zhou, Xin Wang, Wen Yin, Yan Li, Yunlu Guo, Chen Chen, Johannes Boltze, Arthur Liesz, Tim Sparwasser, Daxiang Wen, Weifeng Yu, Peiying Li

**Affiliations:** ^1^ Department of Anesthesiology, Renji Hospital Shanghai Jiao Tong University School of Medicine Shanghai China; ^2^ Key Laboratory of Anesthesiology (Shanghai Jiao Tong University) Ministry of Education Shanghai China; ^3^ School of Life Sciences University of Warwick Coventry UK; ^4^ Institute for Stroke and Dementia Research (ISD), University Hospital LMU Munich Munich Germany; ^5^ Munich Cluster for Systems Neurology (SyNergy) Munich Germany; ^6^ Institute of Medical Microbiology and Hygiene University Medical Center of the Johannes Gutenberg‐University Mainz Mainz Germany; ^7^ Research Center for Immunotherapy (FZI) University Medical Center, Johannes Gutenberg‐University Mainz Mainz Germany; ^8^ Clinical Research Center, Renji Hospital Shanghai Jiao Tong University School of Medicine Shanghai China; ^9^ Outcomes Research Consortium Cleveland Ohio USA

**Keywords:** antigen presenting, CD8^+^ T cell, demyelination, perioperative stroke, RIPK1

## Abstract

**Aim:**

To explore the regulatory mechanisms of microglia‐mediated cytotoxic CD8^+^ T‐cell infiltration in the white matter injury of perioperative stroke (PIS).

**Methods:**

Adult male C57BL/6 mice were subjected to ileocolic bowel resection (ICR) 24 h prior to permanent distant middle cerebral artery occlusion (dMCAO) to establish model PIS. White matter injury, functional outcomes, peripheral immune cell infiltration, and microglia phenotype were assessed up to 28 days after dMCAO using behavioral phenotyping, immunofluorescence staining, transmission electron microscopy, western blot, and FACS analysis.

**Results:**

We found surgery aggravated white matter injury and deteriorated sensorimotor deficits up to 28 days following PIS. The PIS mice exhibited significantly increased activation of peripheral and central CD8^+^ T cells, while significantly reduced numbers of mature oligodendrocytes compared to IS mice. Neutralizing CD8^+^ T cells partly reversed the aggravated demyelination following PIS. Pharmacological blockage or genetic deletion of receptor‐interacting protein kinase 1 (RIPK1) activity could alleviate CD8^+^ T‐cell infiltration and demyelination in PIS mice.

**Conclusion:**

Surgery exacerbates demyelination and worsens neurological function by promoting infiltration of CD8^+^ T cells and microglia necroptosis, suggesting that modulating interactions of CD8^+^ T cells and microglia could be a novel therapeutic target of long‐term neurological deficits of PIS.

## Graphical abstract

Perioperative stroke (PIS) exacerbates post‐stroke demyelination and worsens neurological functions by promoting infiltration of CD8^+^ T cells and microglia necroptosis in experimental animal models. Pharmacological blockage or genetic deletion of receptor‐interacting protein kinase 1 activity could partly reverse demyelination injury, suggesting a novel therapeutic target for the PIS treatment.

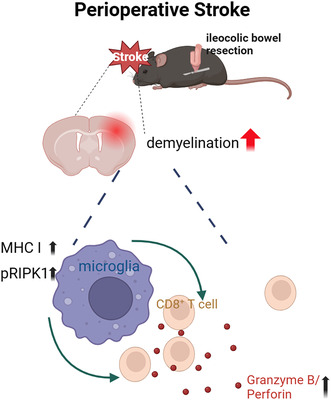

## 1 INTRODUCTION

Perioperative stroke (PIS) is a devastating complication of surgery with increased mortality and morbidity compared to ischemic stroke (IS) alone^1^ and its prevalence is often underestimated after non‐cardiac, non‐neurological surgery.^2^, ^3^ PIS incidence varies depending on the surgical type, study design, and diagnostic criteria, ranging from 0.05% to 7.4%.^1^, ^4^, ^5^, ^6^, ^7^, ^8^ Ischemic stroke accounts for a considerably higher proportion (~95%) and less than 5% of PIS patients benefit from thrombolysis due to the narrow therapeutic window, delayed diagnosis, and high risks of hemorrhage,^9^, ^10^, ^11^ thus a permanent ischemia animal model (dMCAO) was chosen to mimic PIS condition for long‐term investigations. Considering the high annual volume of abdominal surgery globally,^12^, ^13^ and the obvious higher rate (0.7%) of PIS for hemicolectomy among non‐cardiac, non‐neurological surgery^5^; therefore, ileocolic bowel resection (ICR) was used to represent a major intra‐abdominal surgical procedure. Taken together, the authors established “ICR + dMCAO” as a PIS animal model for subsequent experiments.

Understanding the mechanisms underlying exacerbated long‐term neurological deficits following PIS is crucial for the development of evidence‐based strategies to improve outcomes for PIS patients. Recent investigations have identified that macrophage migration inhibitory factor (MIF)‐loaded myeloid cells induced endothelial cell death and CD8^+^ T lymphocyte‐mediated neurotoxicity under perioperative conditions, providing promising immunotherapy targets.^14^, ^15^ Perioperative factors significantly affect the immune system of patients,^16^ resulting in an acute systemic inflammatory response that may amplify the pro‐inflammatory signaling after stroke.

Recently, white matter injury has been increasingly recognized as a major contributor to poor neurological function in both stroke animals and patients.^17^, ^18^ In turn, promoting white matter regeneration and repair has been reported to benefit the structural and functional recovery after stroke.^19^ Previous studies demonstrated that cytotoxic CD8^+^ T cells play a detrimental role in demyelinating injury after experimental stroke or autoimmune encephalomyelitis in mice.^20^, ^21^ The receptor‐interacting protein kinase 1 (RIPK1)‐mediated neuroinflammation is involved in multiple neurodegenerative diseases, leading to demyelination.^22^ Microglia were identified as susceptible to RIPK1‐mediated cell death, which causes a detrimental neuroinflammatory program contributing to the neurodegenerative environment.^23^ However, the complex influences interactions of immune responses on white matter integrity after perioperative stroke remains unclear.

In the current study, we find that surgery exacerbates post‐stroke demyelination and worsens neurological function by promoting infiltration of CD8^+^ T cells and the RIPK1 activation in microglia is critical for the enhanced CD8^+^ T‐cell activation in PIS. Thus, we propose that pharmacological inhibition of RIPK1 could serve as a therapeutic strategy to alleviate CD8^+^ T‐cell infiltration and demyelination injury following PIS.

## 2 MATERIALS AND METHODS

### 2.1 Animals

Adult age‐matched (10‐12 weeks) male C57BL/6 mice were obtained from Shanghai SLAC Laboratory Animals and were kept in conventional laboratory conditions (22°C and a 12‐h light‐dark cycle with free access to food and water). All animal experiments were approved by the Renji Hospital Institutional Animal Care and Use Committee and carried out in accordance with the Institutional Guide for the Care and Use of Laboratory Animals. Randomization and allocation were performed.

### 2.2 Murine focal cerebral ischemia, ileocolic bowel resection, and parabiosis models

Adult male mice were randomly assigned to control, surgery‐only, sham, IS, and PIS groups. Male mice were subjected to distal middle cerebral artery occlusion (dMCAO) as previously described.^24^ Cerebral blood flow was measured using a laser Doppler flowmeter to confirm the vascular occlusion. Animals with a regional cerebral blood flow reduction lower than 30% of pre‐ischemia baseline levels immediately after dMCAO were excluded from further experiments. Sham mice were similarly anesthetized and operated without MCA cauterization. For the PIS model, mice were first anesthetized using 2% enflurane inhalation, followed by ileocolic bowel resection (ICR)^25^ 24 h prior to dMCAO as above described. We used dMCAO alone as the control paradigm (IS mice). Control mice were naïve mice without ICR and dMCAO surgery. Surgery‐only mice were only subjected to ICR without dMCAO. No significant surgery‐induced bacterial infection or decreased long‐term stable nutrition status was monitored in PIS mice (Figures S7 and S8).

Parabiosis was conducted by connecting mice through the elbow, knee joints, and skin.^26^ The parabiosis will then be subjected to PIS or IS model 14 days after parabiosis surgery. Mice were allowed to recover spontaneously from anesthesia during warm conditions. Rectal temperature was maintained at 37 ± 0.5°C via a thermal blanket throughout the above surgical performances. Mice were monitored every morning post‐operatively to ensure their well‐being. If any signs of discomfort or distress were observed, an additional subcutaneous dose of meloxicam (5 mg/kg) was administered. Surgery animals with severe complications were excluded from further experiments. All animals were euthanized using intraperitoneally sodium pentobarbital injection (100 mg/kg) at each timepoint of tissue collection or suffering complications at the anastomosis. Animal data reporting followed the ARRIVE 2.0 guidelines.^27^ The numbers of animals are summarized in Table S1.

### 2.3 Neurofunctional tests

Motor and sensory function tests were conducted 1 day before and 1‐28 days after dMCAO in both PIS and IS mice. Sensorimotor deficits were assessed using the well‐established modified Garcia score, the foot fault test, and corner‐turning test.^28^, ^29^, ^30^ The modified Garcia score involved scoring on a scale of 0 to 3, with a maximal score of 15. The tests included: (1) body proprioception, (2) climbing, (3) forelimb walking, (4) lateral turning, and (5) limb symmetry. Cognitive and memory deficits were evaluated using the Morris water maze test.^31^

### 2.4 Immunofluorescence staining, image acquisition, and analysis

The brain tissues were collected through trans‐cardiac perfusion with ice‐cold phosphate‐buffered saline (PBS) and then fixed with 4% paraformaldehyde (PFA) after euthanization via sodium pentobarbital. After dehydration, the tissues were embedded in O.C.T compound and cut into 25‐μm sections for immunofluorescence staining. A detailed description of staining, imaging processing, and analysis was included in the supplementary methods. All the antibodies are summarized in Table S2.

### 2.5 In vivo depletion of CD8 ^+^ T cells

We injected CD8α‐specific (15 mg/kg, clone 53‐6.72; BioXCell) monoclonal antibody or isotype antibody (IgG, clone 2A3; BioXCell) diluted in sterile PBS intraperitoneally 24 h before dMCAO or 2 h before ICR surgery.^20^ Adult male mice were then randomly allocated to IgG + IS, anti‐CD8 + IS, IgG + PIS, and anti‐CD8 + PIS groups. Successful depletion was determined by the presence of <5% CD8^+^ T cells in the CD3^+^ cell population.

### 2.6 In vivo inhibition of RIPK1

RIPK1 inhibitor R‐7‐Cl O‐necrostatin‐1 (Nec‐1 s) (10 mg/kg) or Vehicle (1% DMSO) were intravenously injected 2 h before dMCAO in PIS mice.^15^

### 2.7 Statistical analysis

All statistical analyses were performed using GraphPad Prism 7 software. All data are expressed as mean ± SD. Sample sizes for animal research were determined by power calculations for the primary parameter with mean differences and standard deviations based on prior studies and previous literature (power 80%, α 0.05).^15^ The Shapiro‐Wilk normality test was initially performed on all data sets. Differences between groups were compared using a two‐tailed unpaired Student *t*‐test. One‐way analysis of variance (ANOVA) was used for the comparison among three or four groups with normal distributions. Differences in means across groups with repeated measurements over time were analyzed via two‐way ANOVA with repeated measures following the post‐hoc Bonferroni test. Animal survival rates were compared using Kaplan‐Meier survival analysis. Statistical differences were considered to be significant when *p* < 0.05.

## 3 RESULTS

### 3.1 Exacerbated long‐term white matter injury and impaired oligodendrocyte progenitor cell maturation in PIS mouse brain

**FIGURE 1:**
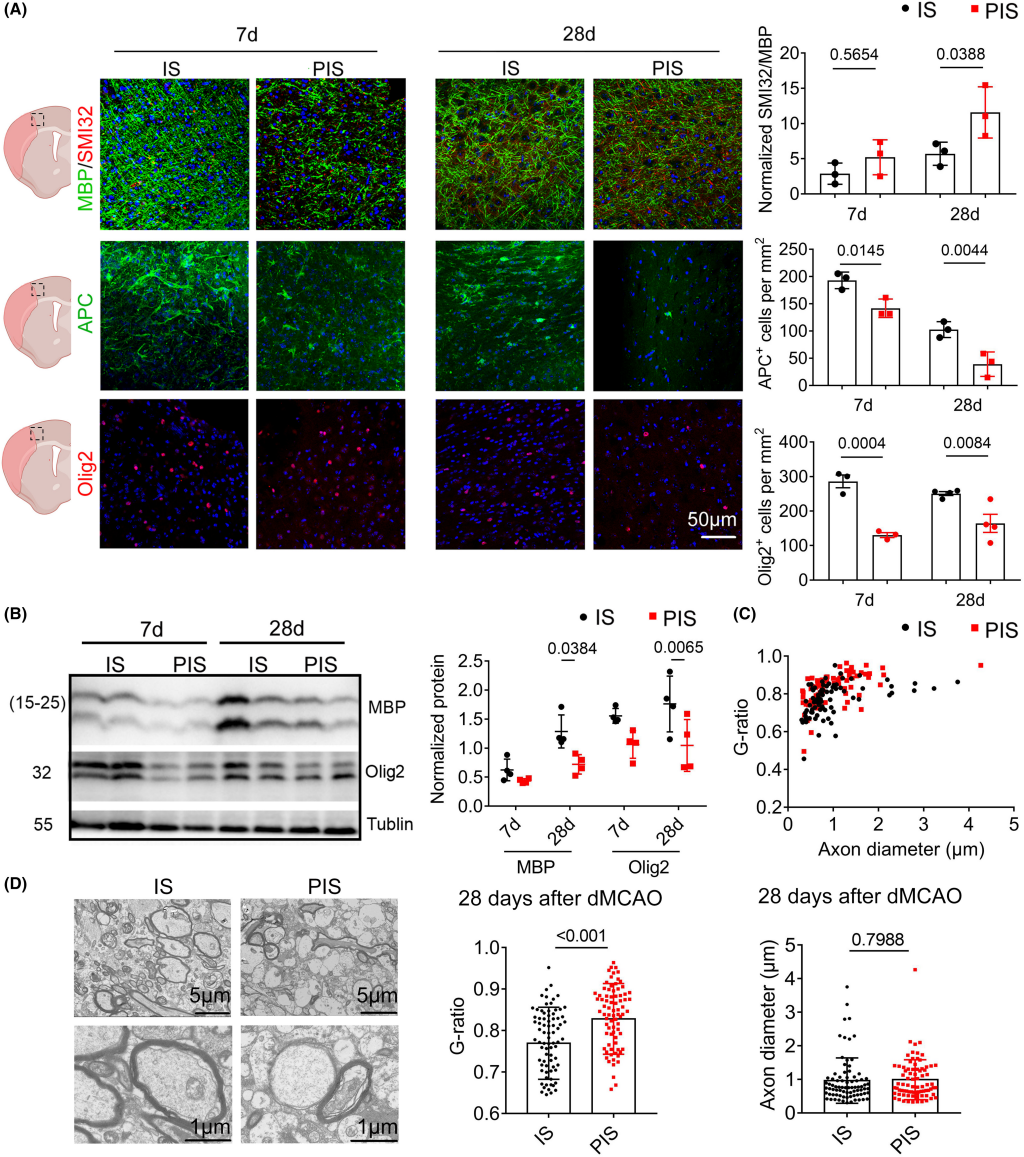
Exacerbated long‐term white matter integrity impairment in PIS mice. (A) Left, representative images of SMI32 and MBP double‐immunostaining, as well as APC and Olig2 staining 7 and 28 days after dMCAO in PIS and IS mice. The dotted box refers to the representative peri‐infarct cortex area of coronal brain section. Scale bar = 50 μm. 4′, 6‐diamidino‐2‐phenylindole (DAPI) was used for nuclear staining. Right, increased ratio of SMI32 (red) and MBP (green) staining intensities, downregulated positive cells of APC and Olig2 in the peri‐ischemic cortex 7 and 28 days after dMCAO in PIS mice. *n* = 3–4 per group; two‐way ANOVA, Bonferroni post‐hoc correction. (B) Decreased levels of MBP and Olig2 28 days after dMCAO in PIS mice compared to IS mice measured by Western blot. *n* = 4 per group; two‐way ANOVA, Bonferroni post‐hoc correction. (C) G‐ratio analysis of remyelinated axons and corresponding axonal diameter. (D) Left, electron microscopy micrographs of cortical lesions 28 days after dMCAO showed reduced numbers of remyelinated axons in PIS mice compared to IS mice. Upper/lower scale bar = 5/1 μm, respectively. Middle, scatter plot showing the increased overall G‐ratio in PIS mice compared to IS mice. Right, similar axon diameters in both PIS and IS mice. *n* = 80 axons from three mice per group; unpaired *t*‐test.

Our group has identified the acute endothelial cell death during perioperative condition,^15^ we further evaluated the long‐term white matter integrity in PIS mice. SMI‐32 antibodies bind to the non‐phosphorylated epitope of neurofilament H, indicating demyelinating injury. The peri‐infarct cortex of PIS mice showed enhanced SMI‐32 immunoreactivity and reduced MBP (myelin basic protein) immunostaining, resulting in an increased ratio of SMI‐32 to MBP compared to IS‐only mice 28 days after dMCAO (Figure 1A). We also found a significant reduction in the number of mature oligodendrocytes stained with anti‐adenomatous polyposis coli (APC^+^) and oligodendrocyte transcription factor 2 (Olig2^+^) developmental oligodendrocytes in PIS mice compared to IS mice at 7 and 28 days after dMCAO (Figure 1A). Besides, we analyzed the contents of white matter differentiation markers related to later developmental steps (MBP, Olig2) by western blot in the peri‐ischemic cortex and observed a significant decrease in MBP and Olig2 expressions in PIS mice compared to that of IS mice at 28 days (Figure 1B, Figure S6). Moreover, the G‐ratios were significantly increased in the PIS group, indicating thinner myelin and reduced remyelinated axons in the lesioned cortex (Figure 1C,D). However, axonal diameters were not significantly different between the groups (Figure 1D). Similarly, we also found that PIS mice exhibited significantly increased infarct volumes 3 days after dMCAO compared to IS mice (Figure S1B). Laser speckle imaging revealed that cerebral blood flow in PIS mice was significantly lower than in IS mice 3 days following dMCAO, whereas CBF was comparable between the two groups at 7, 14, and 28 days following dMCAO (Figure S1A). The percentage of survival 28 days after dMCAO was significantly lower in the PIS group compared to those of the IS or surgery‐only group (Figure S1D). There was no significant difference in neuronal tissue loss or the number of morphologically viable neurons, as measured by MAP2 and NeuN immunostaining in the peri‐infarct cortex 28 days after dMCAO between the two groups (Figure S1C,D). Collectively, our data demonstrated that PIS mice exhibited significantly exacerbated long‐term demyelination and impaired oligodendrocyte progenitor cells (OPC) maturation after stroke.

### 3.2 PIS mice exhibit deteriorated sensorimotor and memory functions after dMCAO

It has been suggested that white matter injury after stroke is robustly correlated with long‐term functional deficits.^32^, ^33^ Therefore, we further evaluated the influence of surgery on long‐term functional outcomes at various time points after dMCAO by the modified Garcia score (1‐28 days), foot fault test (1‐28 days), and Morris water maze (19‐22 days). We found that PIS mice exhibited significantly lower total neurological scores compared to IS mice (Figure S2A). PIS mice also experienced significantly deteriorated gait, as reflected by the increased forelimb and total foot fault errors compared to IS mice (Figure S2B). Furthermore, in the Morris Water Maze, PIS mice spent significantly less time in the target quadrant when the platform had been removed after the learning trials (impaired memory) compared to IS mice (Figure S2C,D). However, the PIS and IS mice discovered the hidden platform at comparable times (similar spatial learning) during the training period. Similar swim speeds were found among those three groups (Figure S2D).

The above findings in PIS mice demonstrated sustained surgery‐related sensorimotor and memory deficits after dMCAO.

### 3.3 Enhanced persistent activation of local CD8 ^+^ T cells in the PIS mouse brain

**FIGURE 2:**
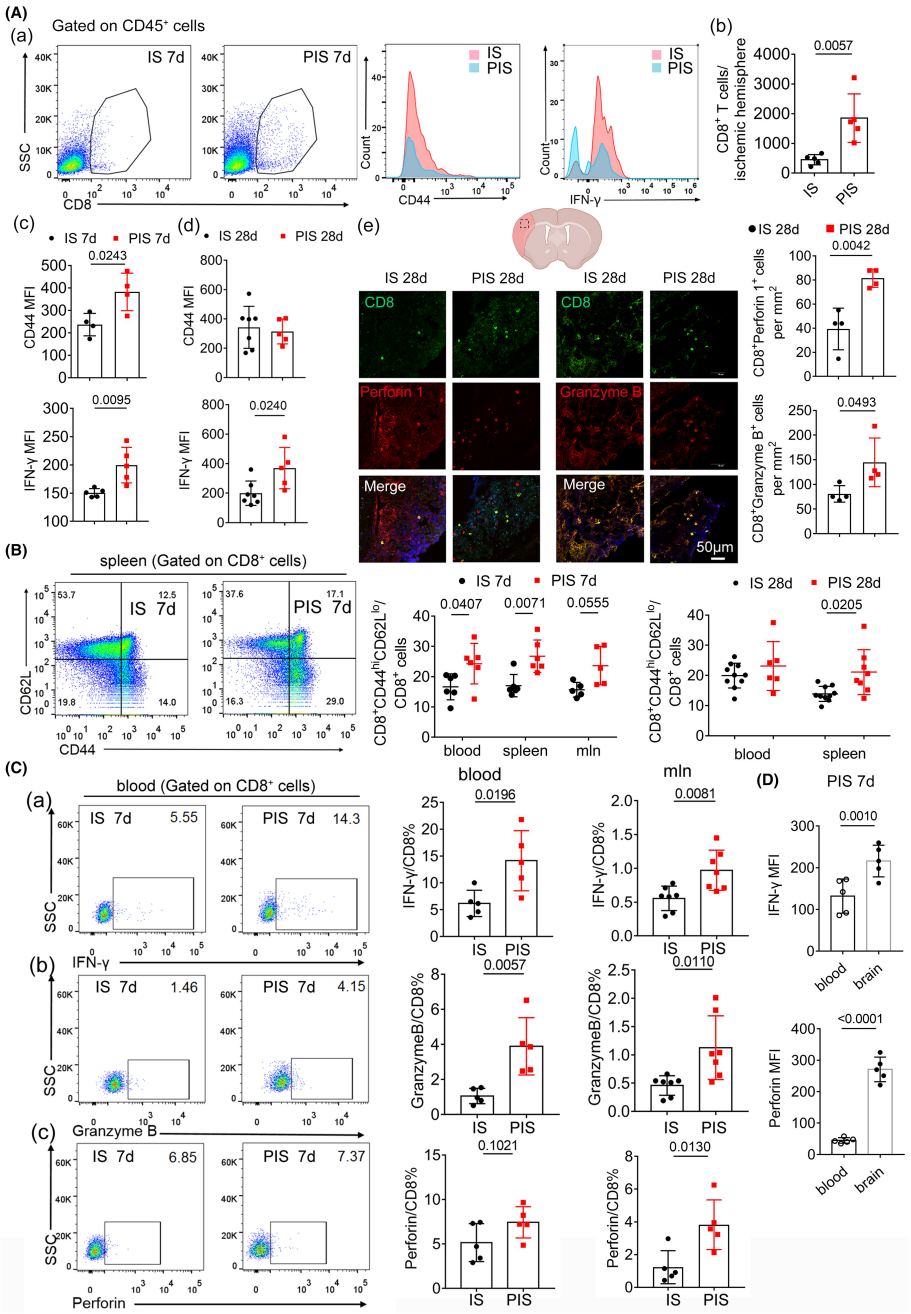
Enhanced activation of CD8^+^ T cells in the ischemic brain of PIS mice. (A) (a–d) Representative flow cytometry images and quantification of CD8^+^ T cells infiltrating the ischemic hemisphere, as well as MFI of CD44, IFN‐γ CD8^+^ T cells in PIS and IS mice 7 days after dMCAO. Increased numbers of CD8^+^ cells, MFI of CD44, and IFN‐γ were identified. *n* = 4–6 per group; unpaired *t*‐test. (e) Representative images and quantification of immunofluorescence staining of CD8^+^ T cells in PIS and IS mice 28 days after dMCAO, scale bar = 50 μm. The dotted box refers to the representative infarct cortex area of coronal brain section. Increased CD8^+^ T cells and Perforin 1^+^CD8^+^ T cells were identified in PIS mice. *n* = 4 per group; unpaired *t*‐test. (B) Percentage of CD8^+^CD44^hi^CD62L^lo^ cells in peripheral blood, spleen, and mesenteric lymph nodes (MLN) were significantly increased in PIS compared to IS mice 7 and 28 days after dMCAO. *n* = 5–6 per group; two‐way ANOVA, Bonferroni post‐hoc correction. (C) The percentage of IFN‐γ (a) and Granzyme B (b) in both peripheral blood and MLN significantly increased in PIS mice compared to IS mice while Perforin (c) only increased in MLN 7 days after dMCAO. *n* = 5–7 per group; unpaired *t*‐test. (D) IFN‐γ and Perforin MFI of peripheral and cerebral CD8^+^ T cells isolated 7 days after dMCAO. *n* = 5–6 per group; unpaired *t*‐test.

Previous research has suggested that infiltrated T cells, particularly MBP‐specific CD8^+^ T cells, may exacerbate neuroinflammation following cerebral IS.^34^ Our previous study also found that infiltrated CD8^+^ T cells were detrimental to demyelination after IS.^20^ In this study, we further observed that PIS mice exhibited significantly higher absolute counts of brain‐invading CD8^+^ T cells and increased expression of CD44, IFN‐γ, and Granzyme B in CD8^+^ T cells compared to IS mice 7 days after injury. Moreover, increased IFN‐γ expression and absolute number of brain‐invading CD8^+^ T cells mice were detected in PIS mice up to 28 days after dMCAO, while the mean fluorescent intensity (MFI) of CD44 of the CD8^+^ T cells was comparable between the two groups (Figure 2A; Figure S3A,B). Immunofluorescence staining also showed persistent increased numbers of CD8^+^, Perforin 1^+^CD8^+^, and Granzyme B^+^CD8^+^ cells in PIS mice compared with IS mice until 28 days after dMCAO (Figure 2A). The percentage of activated CD8^+^ T cells (CD8^+^CD44^hi^CD62L^lo^ T cells) was significantly higher in peripheral blood and spleen from PIS compared to IS mice (Figure 2B). Correspondingly, the peripheral CD8^+^ T cells from the blood and mesenteric lymph nodes (MLN) also showed significantly increased expression of IFN‐γ and Granzyme B in PIS mice compared to IS mice. The percentage of Perforin^+^/CD8^+^ T cells in the MLN but not in the blood was significantly increased in the PIS mice compared to IS mice (Figure 2C). Activation of brain‐infiltrated CD8^+^ T cells was significantly reinforced, as evidenced by IFN‐γ and perforin production, compared to those from the periphery 7 days after dMCAO (Figure 2D). Besides, surgery‐only mice also showed a significant augment of Granzyme B^+^CD8^+^ T and CD8^+^CD44^hi^CD62L^lo^ T cells in blood compared to control mice 1 day after ICR surgery (Figure S3E). In contrast, overall immunological milieux including splenic CD4^+^ T cells, Gr1^+^, and B220^+^ cells remained unchanged between IS and PIS groups (Figure S3C,D), enhancing the importance of specific CD8^+^ T cell activation.

In order to further examine the role of peripheral inflammatory factors in the development of enlarged PIS infarcts, we further generated parabiotic animal models allowing blood exchange between surgically conjoined mice to investigate the role of peripheral factors in mediating the outcomes of PIS mice (Figure S4A). We identified increased infarct volume and normalized SMI32/MBP ratio of cortex and EC areas in IS mice paired with ICR mice compared to those paired with non‐ICR mice (Figure S4B,C).

Thus, our findings demonstrated increased numbers and an enhanced pro‐inflammatory state of peripheral and brain‐invading CD8^+^ T cells which were positively correlated to the increased demyelination in PIS mice compared to IS‐only mice.

### 3.4 CD8 ^+^ T cell depletion ameliorates white matter injury and neurodeficits in PIS mice

**FIGURE 3:**
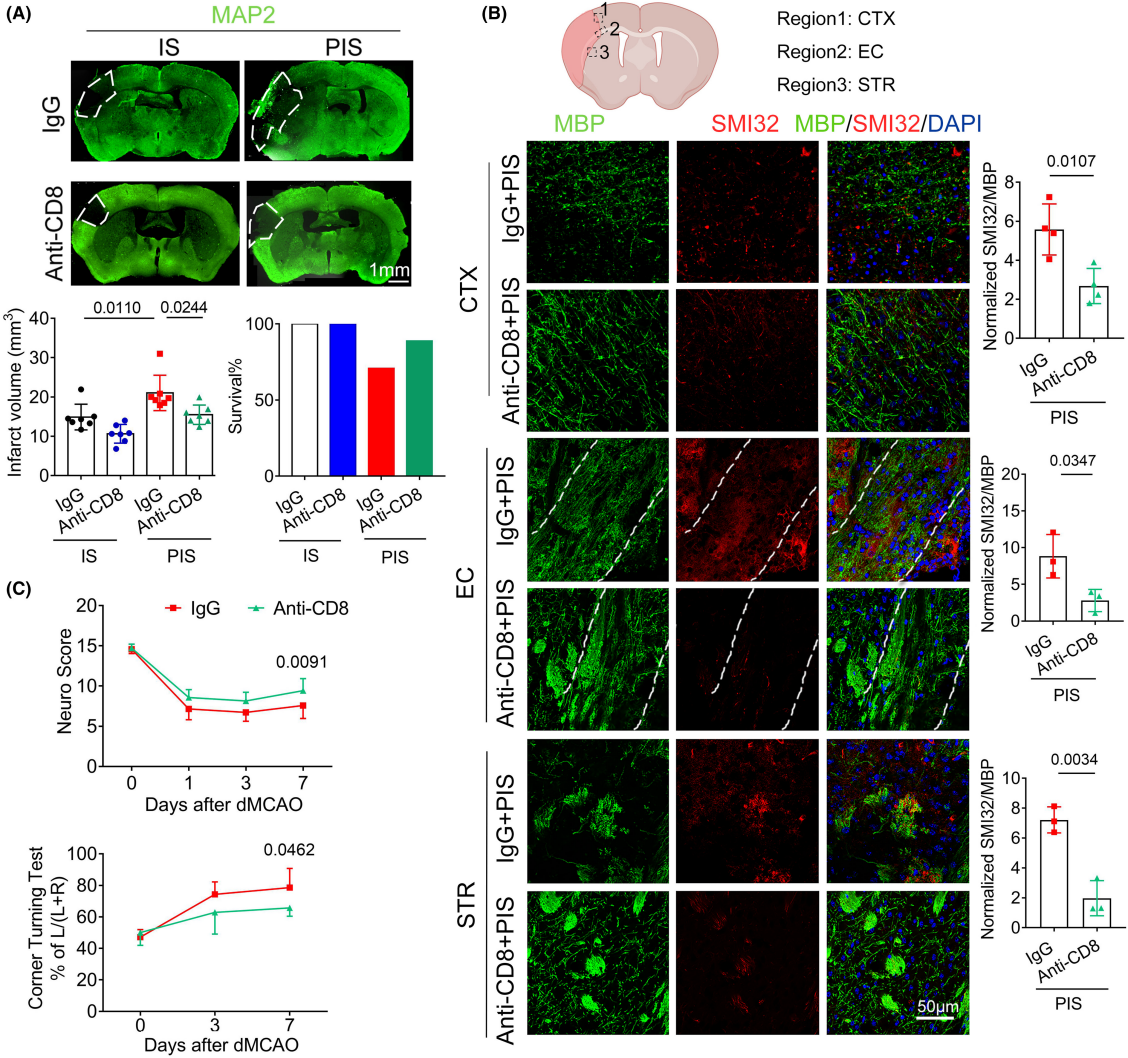
CD8^+^ T cell depletion ameliorates white matter injury and neurodeficits in PIS mice. (A) Infarct volumes 7 days after dMCAO were reduced in PIS mice after CD8^+^ T cell depletion. *n* = 7 per group; one‐way ANOVA, Bonferroni post‐hoc correction. Survival rate was increased in PIS mice after CD8^+^ T cell pre‐depletion. (B) Representative images and normalized quantification of SMI32 and MBP double‐immunostaining in the cortex, external capsule (EC), and striatum (STR) area of IgG‐treated and anti‐CD8‐treated PIS mice. The dotted box refers to the representative peri‐infarct CTX, EC, and STR areas of coronal brain section. *n* = 3 per group; unpaired *t*‐test. (C) Sensorimotor functions were assessed as modified Garcia score and corner test at indicated time points after dMCAO in PIS mice. *n* = 7 per group; two‐way ANOVA with repeated measures, Bonferroni post‐hoc correction.

To investigate the causal relationship between brain‐invading CD8^+^ T cells and aggravated white matter injury in PIS mice, we depleted CD8^+^ T cells by intraperitoneally administering neutralizing antibodies 2 h prior to ICR surgery and demonstrated the efficiency (Figure S9). Consistent with a previous study,^35^ CD8^+^ T cell depletion alleviated infarct volumes compared to IgG‐treated controls 7 days after dMCAO. Furthermore, we observed decreased infarct volumes and increased survival rates in CD8^+^ T cell‐depleted PIS mice 7 days after dMCAO compared to IgG‐treated PIS mice (Figure 3A). With respect to white matter integrity, CD8^+^ T cell depletion significantly reduced the intensity of SMI‐32 immunostaining and the SMI‐32/MBP ratio in the injury‐related cortex, EC, and striatum areas of PIS mice (Figure 3B). Consequently, total neurological scores and postural asymmetries assessed by corner‐turning test were significantly promoted in CD8^+^ T cell‐depleted PIS mice (Figure 3C).

Collectively, these findings suggest that persistently activated CD8^+^ T cells are critical for deteriorated white matter injury observed in PIS mice.

### 3.5 CD8 ^+^ T cell depletion promotes white matter integrity in PIS mice

**FIGURE 4:**
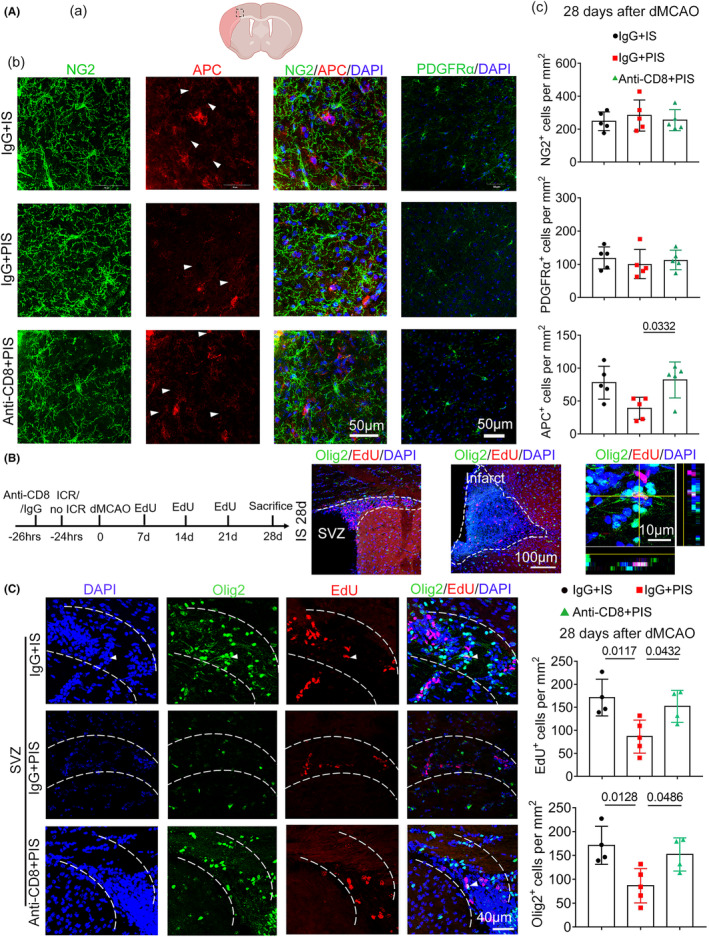
CD8^+^ T cell depletion promotes white matter integrity in PIS mice. (A) (a) The dotted box refers to the representative peri‐infarct cortex area of coronal brain section. (b) Higher magnification of double staining of NG2 (green), PDGFRα (green), and APC (red) cells close to the injured tissue. Nuclear staining with DAPI is shown in blue. Scale bar = 50 μm. Triangle marker refers to APC^+^ mature oligodendrocytes. (c) Quantification of NG2^+^, PDGFRα^+^, and APC^+^ cells. *n* = 5 per group; one‐way ANOVA, Bonferroni post‐hoc correction. (B) Left, experimental design of EdU injection experiments. Middle, representative images of EdU (red) and Olig2 (green) immunostaining 28 days after dMCAO in the subventricular zone (SVZ) and peri‐infarct area of IS mice. Scale bar = 100 μm. Right, higher magnification of double staining with Olig2 (green) and EdU (red) cells. Scale bar = 10 μm. (C) Representative images and quantification of EdU (red) and Olig2 (green) immunostaining 28 days after dMCAO among the SVZ area. Scale bar = 40 μm. Triangle marker refers to Olig2^+^EdU^+^ newly generated oligodendrocytes. *n* = 4–5 per group; one‐way ANOVA, Bonferroni post‐hoc correction.

Remyelination is a critical process involving the recruitment and differentiation of OPCs into mature myelin‐forming oligodendrocytes, which restore white matter integrity and nerve function after CNS diseases.^19^ Therefore, we investigated long‐term OPC responses in PIS mice. We found that there was no difference in the total number of reactive NG2^+^ cells and PDGFRα^+^ cells in IS mice, PIS mice, or CD8‐depleted PIS mice. Notably, the number of APC^+^ mature oligodendrocytes was significantly decreased in PIS mice compared to IS mice, while CD8 depletion reversed this negative effect (Figure 4A), reinforcing the inhibitory effects of CD8^+^ T cells on OPC maturation in PIS mice.

Next, we examined the regeneration of oligodendrocytes by EdU injection and detected the EdU^+^ and Olig2^+^ cells from target subventricular zone (SVZ) and peri‐infarct areas (Figure 4B) in the above groups. The total number of both EdU^+^ newly generated cells and Olig2^+^ oligodendrocytes was significantly decreased in PIS mice compared to IS mice and increased in CD8‐depleted PIS mice compared to the isotype‐treated PIS mice in the subventricular zone (SVZ). However, the majority of Olig2^+^ oligodendrocytes were EdU negative, indicating that oligodendrocytes were rarely regenerated (Figure 4C). In summary, CD8^+^ T cell depletion promoted long‐term white matter integrity in PIS mice.

### 3.6 Increased antigen presentation and necroptosis of microglia in PIS mice

**FIGURE 5:**
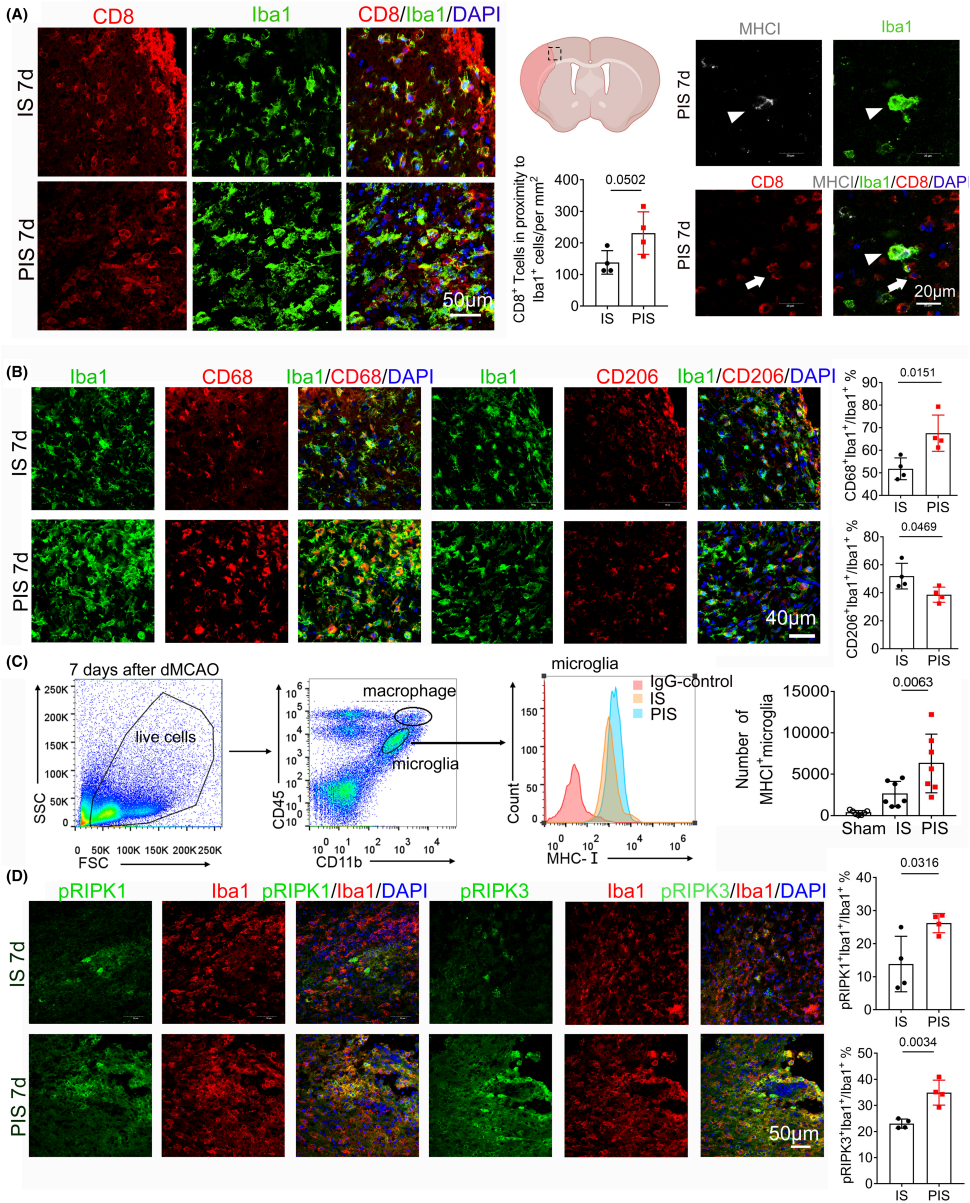
Increased antigen presentation and necroptosis of microglia in PIS mice. (A) Immunofluorescence (Left) and quantification (Middle) of CD8^+^ T cells and Iba1^+^ microglia in proximity in the peri‐ischemic area of IS and PIS mice. The dotted box refers to the representative peri‐infarct cortex area of coronal brain section. Scale bar = 50 μm. Representative image of MHC I^+^Iba1^+^ (triangle marker) cells localized to the CD8^+^ cell (arrow marker) 7 days after dMCAO in IS mice. Scale bar = 20 μm (Right). (B) Representative images and quantification of CD68 (red), CD206 (red), and Iba1 (green) staining 7 days after dMCAO in PIS and IS mice. Scale bar = 40 μm. *n* = 4 per group; unpaired *t*‐test. (C) Representative flow cytometry images of CD45^int^CD11b^+^ microglia and CD45^hi^CD11b^+^ macrophages infiltrated in the ischemic hemisphere, and further analysis of MHC I expression (Right). Quantification of the absolute numbers of MHC I^+^ microglia in Sham, IS, and PIS mice. *n* = 7 per group; one‐way ANOVA, Bonferroni post‐hoc correction. (D) Representative images and quantification of pRIPK1 (green), pRIPK3 (green), and Iba1 (red) staining 7 days after dMCAO in PIS and IS mice. Scale bar = 50 μm. *n* = 4 per group; unpaired *t*‐test.

Microglia and astrocytes are the resident immune cells that surveil the micro‐environment in the brain.^36^ More brain‐invading CD8^+^ T cells were found in close proximity to Iba1^+^ cells 7 days after dMCAO in PIS mice (Figure 5A). We also observed robust microglia/macrophage polarization toward a pro‐inflammatory phenotype (increased CD68^+^Iba1^+^ combined with decreased CD206^+^Iba1^+^) in PIS mice (Figure 5B). To further verify the phenotypic changes of microglia and macrophages after PIS, myeloid cells were first identified by CD45 and CD11b expression using FACS analysis and then differentiated into CD45 high (CD45^hi^) macrophages and intermediate (int) CD45 microglia (CD45^int^ CD11b^+^). CD45^int^ CD11b^+^ microglia in the PIS mouse brain showed significantly upregulated expression of major histocompatibility complex class I (MHC I), major histocompatibility complex class II (MHC II) and costimulatory molecules CD86 compared to those in IS mice 7 days after dMCAO (Figure 5C; Figure S5A). Microglia in the ischemic cortex of PIS mice brain also exhibited an increased number/percentage of amoeboid‐shaped MHC I^+^Iba1^+^ phenotype compared to IS mice, indicating augmented ability to active CD8^+^ T cells (Figure S5B). Biomarkers for necroptosis, such as pRIPK1 and pRIPK3 in Iba1^+^ cells were further examined. The percentage of necroptotic Iba1^+^ cells was increased in PIS mice as well (Figure 5D). Furthermore, no differences were detected in the astrocytic response which was measured by GFAP^+^ cells of IS and PIS mice at 7 or 28 days after dMCAO (Figure S5C). In order to examine the effects of surgery on the glial response, surgery‐only mice showed a significantly increased number of activated Iba1^+^ cells 1 day after surgery compared with control mice, while no significant differences of GFAP^+^ cells were detected between surgery‐only and control mice at 1 or 4 days after ICR surgery (Figure S5D). FACS analysis revealed similarly increased expressions of MHC I, MHC II, and CD86 in microglia among surgery‐only mice compared to the control mice 1 day after ICR operation, suggesting the upregulated antigen‐presenting ability of microglia (Figure S5E). Taken together, the above data suggested PIS complicated antigen presentation and necroptosis of microglia.

### 3.7 Inhibition of RIPK1 reduces CD8 ^+^ T‐cell infiltration and white matter injury in PIS mice

**FIGURE 6:**
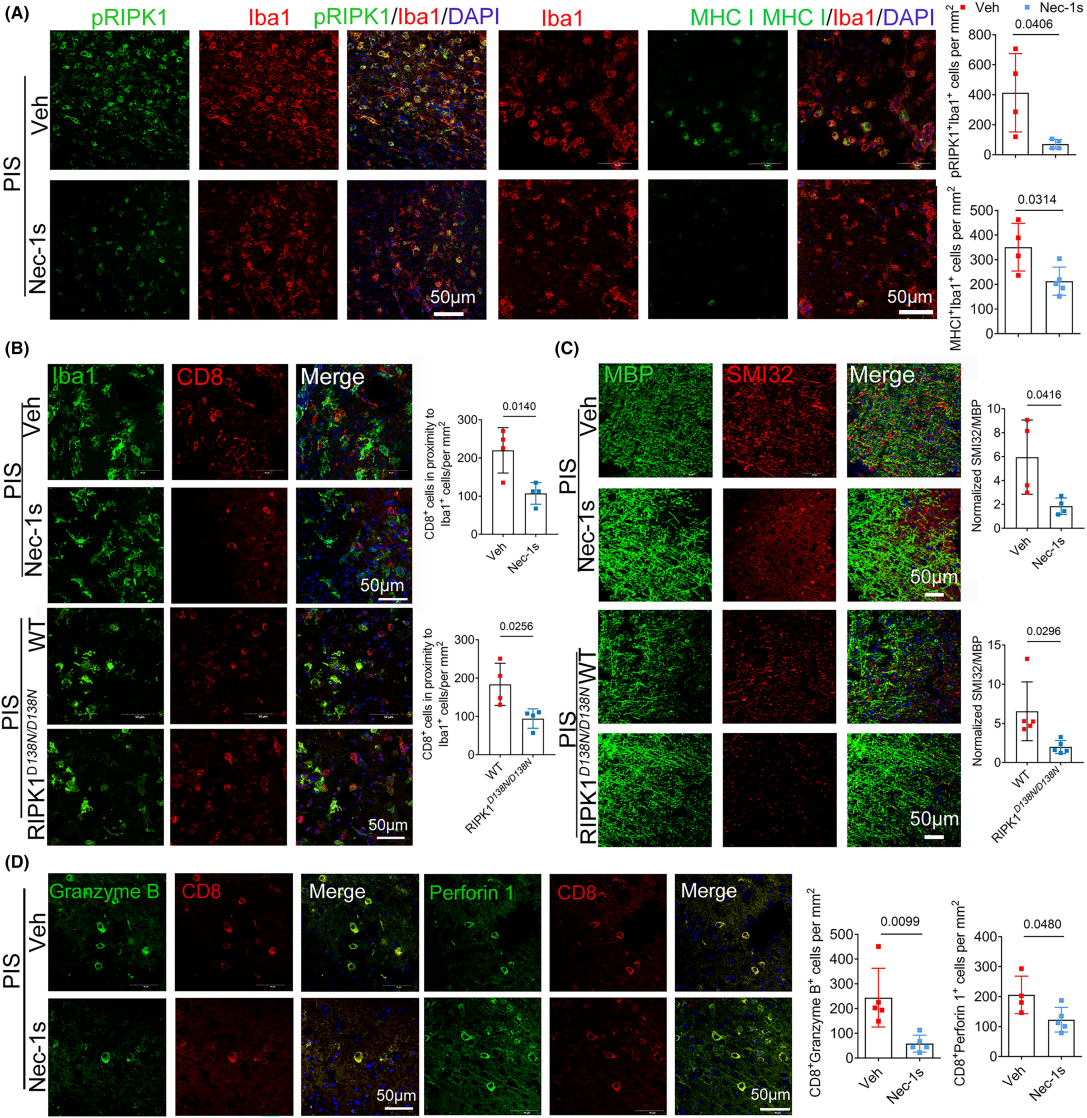
Inhibition of RIPK1 reduces CD8^+^ T‐cell infiltration and white matter injury in PIS mice. (A) Representative images (Left) and quantification (Right) of pRIPK1^+^Iba1^+^ cells and MHC I^+^Iba1^+^ cells in peri‐infarct areas of indicated groups. Scale bar = 50 μm. *n* = 4–5 per group. (B) Representative images (Left) and quantification (Right) of decreased number of CD8^+^ cells in proximity to Iba1^+^ cells in peri‐infarct cortex from Nec‐1s treated and RIPK1^*D138N/D138N*^ PIS mice 3 days after dMCAO. (C) Representative images of SMI32 and MBP double‐immunostaining (Left) and quantification (Right) of normalized SMI32/MBP of indicated groups. Scale bar = 50 μm. *n* = 4 per group, unpaired *t*‐test. (D) Representative images (Left) and quantification (Right) of decreased number of CD8^+^ Perforin 1^+^ cells and CD8^+^ Granzyme B^+^ cells in peri‐infarct areas after Nec‐1s treatment. Scale bar = 50 μm. *n* = 4–5 per group.

To evaluate the functions of RIPK1 on white matter injury during PIS, we administered RIPK1 inhibitor Nec‐1s 2 h before dMCAO in PIS mice according to our previous study and subjected RIPK1 kinase‐dead knockin (RIPK1^*D138N/D138N*^) mice to PIS. Our previous work has revealed reduced infarct volumes and improved neurological functions in Nec‐1s‐treated PIS mice.^15^ Consistently, Nec‐1s treatment markedly reduced pRIPK1^+^Iba1^+^ necroptotic cells and MHC I^+^Iba1^+^ cells in the peri‐infarct cortex of PIS mice as compared to the Veh group (Figure 6A). Meanwhile, the numbers of brain‐invading cytotoxic CD8^+^ T cells were significantly reduced in Nec‐1s‐treated group and RIPK1^*D138N/D138N*^ PIS mice (Figure 6B,D). Consistently, the disruption of white matter was partly restored with Nec‐1s treatment RIPK1^*D138N/D138N*^ mice in PIS (Figure 6C). These data suggest that targeting RIPK1 kinase activity could serve as a promising candidate to inhibit CD8^+^ T‐cell infiltration and ameliorate white matter injury in PIS condition.

## 4 DISCUSSION

PIS is a significant surgical complication which is detrimental to brain health and can cause long‐term neurological deficits. The underlying mechanisms that contribute to poor clinical outcomes of PIS remain largely unexplored. In this study, we show that surgery significantly exacerbates long‐term behavioral deficits and demyelination up to 28 days after PIS. CD8^+^ T cells are profoundly activated both in the periphery and ischemic brain of PIS mice and play critical roles in aggravating the demyelination following PIS. Meanwhile, activation of RIPK1 in microglia of PIS mice was correlated with infiltration of CD8^+^ T cells and demyelinating injury. The current finding highlights a previously unrecognized mechanism underlying the neurological deterioration after PIS and proposes inhibition of RIPK1 to modulate CD8^+^ T cells as a potential therapeutic target in PIS.

Pre‐existing conditions, such as infections and tumors, can result in abnormal vasculature leading the increased risks of both infarction and hemorrhage.^11^ Cellular and humoral elements of the innate immune system are critical in bacterial infection progression and resolution. Myeloid cells (Mϕ, monocytes, dendritic cells, and mast cells) could recognize conserved microbial products, causing the release of pro‐inflammatory mediators, pathogens elimination, antigen presentation, and neutrophil recruitment.^37^ Recent study demonstrated that bacterial infection could trigger assembly of the complex of mechanical sensor Piezo1 and Toll‐like receptor‐4 (TLR4), and then augment phagocytosis, mitochondrion‐phagosomal ROS production in macrophage.^38^ In the acute phase after stroke, a powerful double‐edged innate immune response and systemic immunity were activated by the danger signals in the circulation at the same time. In chronic phase, macrophages may be protective and set the stage for post‐ischemic repair processes.^39^ Appropriate management of innate immune response in bacterial infections is crucial to the final perioperative stroke outcome.

It has been shown that OPCs can migrate to the peri‐injury area and differentiate into mature oligodendrocytes to rebuild the white matter after CNS injury.^40^, ^41^ Promoting the maturation of OPCs could facilitate long‐term functional recovery after experimental stroke.^33^, ^42^ The demyelination and long‐term remyelination PIS mice were partly reversed by pre‐stroke deletion of CD8^+^ T cells. Previous studies in multiple sclerosis have shown that CD8^+^ T cells play pathogenic roles in causing demyelinating lesions through their cytotoxic activity and production of proinflammatory cytokines.^43^ CNS‐infiltrating T cells mainly express CD8, Granzyme B and exhibit oligoclonal expansion.^44^ Notably, we found highly activated CD8^+^ T cells expressing more proinflammatory cytokines both peripherally and centrally 7 days after stroke in PIS mice.

T lymphocytes are known to interact with microglia and regulate the inflammatory cascade in the CNS.^45^, ^46^ Microglia‐derived CCL2/CCL8 chemokines could recruit CD8^+^ T cells in non‐infectious brain diseases.^47^ CD8^+^ T cells infiltrated in the CNS after stroke may acquire features of tissue‐memory cells, which can be locally reactivated.^48^ Microglia could serve as APCs and enhance OT‐1 T cell proliferation in vitro.^49^ However, other studies have indicated that the capacity of microglia to present antigens to T cells is limited.^50^ These previous studies focused on effects of APCs on CD4^+^ T cells, and less is known about the interplay between microglia and CD8^+^ T cells in MS and IS. In our study, we identified increased MHC I^+^Iba1^+^ cells closely localized to CD8^+^ T cells in PIS, indicating interactions between microglia and CD8^+^ T cells.

The immune system could initiate phenotypic shift and dysfunction in response to various surgery‐related risk factors, such as mental stress.^51^, ^52^, ^53^ Stress exposure causes purine metabolic disorder‐induced dysfunction of T lymphocyte and mood behavior.^54^ Phenotype, proliferation, and functional properties of microglia were altered in the emotionally associated brain regions upon psychological stress exposure,^55^ while suppression of deleterious M1 phenotype relieves chronic stress‐induced depressive symptoms.^56^

Our group previously revealed MIF induced increased endothelial cell necroptosis through RIPK1‐dependent pathway in PIS mice.^15^ We further confirmed that the necroptotic markers (pRIPK1 and pRIPK3) were both upregulated in Iba1^+^ cells. Necroptosis is a key regulator of microglia activation in multiple neuroinflammatory conditions.^57^, ^58^ The pharmacological inhibition and genetic deletion of RIPK1 alleviated cytotoxic CD8^+^ T cell infiltration and protected white matter integrity in PIS mice, potentially providing therapeutic utilities for PIS.

In summary, we demonstrated that PIS mice showed increased activation of RIPK in microglia and infiltration of CD8^+^ T cells, leading to reduced white matter integrity, thus exacerbating the neurological deficit after stroke. The current study suggested that targeting RIPK1 to regulate CD8^+^ T cells could be a novel therapeutic target for the immune modulation of PIS.

## AUTHOR CONTRIBUTIONS

YZ, XW, and WY performed ICR surgeries and dCMAO models, behavioral tests, immunofluorescence staining, FACS analysis, and western blot, collected the data, and wrote the manuscript. YL, CC, and YG performed the Edu injections and parabiotic animal models. PL, WY, and DW contributed to critical discussion, supervised the project, and revised the manuscript. AL, TS, and JB had critical comments on the manuscript. All authors read and approved the final manuscript.

## FUNDING INFORMATION

The work is supported by National Natural Science Foundation of China (81971223, 91957111, 81971096, U22A20295, 82061130224, 82101370, 82001381, 82101290, 81970478), Shanghai Engineering Research Center of Peri‐operative Organ Support and Function Preservation (20DZ2254200). P.L. is supported by New Frontier Technology Joint Research sponsored by Shanghai Shenkang Hospital Development Center (SHDC12019102), Shanghai Municipal Education Commission‐Gaofeng Clinical Medical Grant Support (20181805), “Shuguang Program” supported by Shanghai Education Development Foundation and Shanghai Municipal Education Commission (20SG17), and “Shanghai Outstanding Academic Leaders Program” and “Sino‐German Collaborative Program” from Shanghai Municipal Science and Technology Committee (20XD1422400, 23410711500) and the Institutional Clinical Research Program (PYII20‐03). P.L. and J.B. are supported by a Newton Advanced Fellowship grant provided by the UK Academy of Medical Sciences (NAF\R11\1010). P.L. is also supported by the Innovative Research Team of High‐level Local Universities in Shanghai (SHSMU‐ZLCX20211602). A.L. is funded by the Vascular Dementia Research Foundation, the European Research Council (ERC‐StGs 802305) and the German Research Foundation (DFG) under Germany’s Excellence Strategy (EXC 2145 SyNergy – ID 390857198 and FOR 2879 – ID 405358801). W.Y., T.S., P.L., and A.L. are supported by the Sino‐German Mobility Program (M‐0351, M‐0671). X. W. is supported by China Postdoctoral Science of Foundation (2022M712118).

## CONFLICT OF INTEREST STATEMENT

Peiying Li is Associate Editor of CNS Neuroscience and Therapeutics and a corresponding of this article. Johannes Boltze is an Editorial Board member of CNS Neuroscience and Therapeutics and a co‐author of this article. To minimize bias, they were excluded from all editorial decision‐making related to the acceptance of this article for publication.

## CONSENT FOR PUBLICATION

The authors declared that the results/data/figures in this manuscript have not been published elsewhere, nor are they under consideration by another publisher.

## Supporting information


Appendix S1.

## Data Availability

All data included in the experiment are presented in the manuscript and/or the Supplementary Materials. All the original data are available upon request.
