# Supplementary material for: Perioperative stroke deteriorates white matter integrity by enhancing cytotoxic CD8 + T‐cell activation
Source: CNS Neurosci Ther. 2024 Jul 7;30(7):e14747. doi: 10.1111/cns.14747 (PMC11227991; doi:10.1111/cns.14747)
Supplement: Supplementary file 1 — Appendix S1. [file CNS-30-e14747-s001.docx]

**Title: Perioperative stroke deteriorates white matter integrity by enhancing cytotoxic CD8^+^ T cell activation**

Yuxi Zhou^1,2#^, Xin Wang^1,2#^, Wen Yin^1,2#^, Yan Li^1,2^, Yunlu Guo^1,2^, Chen Chen^1,2^, Johannes Boltze^3^, Arthur Liesz^4,5^, Tim Sparwasser^6,7^, Daxiang Wen^1,2*^, Weifeng Yu^1,2*^, Peiying Li^1,2,8,9*^

^1^Department of Anesthesiology, Renji Hospital, Shanghai Jiao Tong University School of Medicine, Shanghai, 200127, China

^2^Key Laboratory of Anesthesiology, Shanghai Jiao Tong University, Ministry of Education, China

^3^School of Life Sciences, University of Warwick, Coventry CV4 7AL, United Kingdom

^4^Institute for Stroke and Dementia Research (ISD), University Hospital, LMU Munich, Germany.

^5^Munich Cluster for Systems Neurology (SyNergy), Germany (A.L.)

^6^Institute of Medical Microbiology and Hygiene, University Medical Center of the Johannes Gutenberg-University Mainz, Mainz, Germany

^7^Research Center for Immunotherapy (FZI), University Medical Center, Johannes Gutenberg-University Mainz, Mainz, Germany

^8^Clinical Research Center, Renji Hospital, Shanghai Jiao Tong University School of Medicine, Shanghai, 200127, China

^9^Outcomes Research Consortium, Cleveland, OH, United States

Short Running Title: CD8^+^ T cells induced demyelination of perioperative stroke

*Correspondence:

Dr. Peiying Li, MD, PhD.

Department of Anesthesiology; Clinical Research Center

Renji Hospital, Shanghai Jiao Tong University School of Medicine

Rm 327; Building 1, 1630 Dongfang Rd, Shanghai 200127, China

Email: [peiyingli.md@gmail.com](mailto:peiyingli.md@gmail.com)

Or

Weifeng Yu, MD, PhD

Department of Anesthesiology

Renji Hospital, Shanghai Jiao Tong University School of Medicine

160 Pujian Rd, Shanghai 200127, China

Email: [ywf808@yeah.net](mailto:ywf808@yeah.net)

Or

Daxiang Wen, MD, PhD

Department of Anesthesiology

Renji Hospital, Shanghai Jiao Tong University School of Medicine

160 Pujian Rd, Shanghai 200127, China

Email: [wdxrwj@126.com](mailto:wdxrwj@126.com)

# Yuxi Zhou, Xin Wang and Wen Yin contributed equally to this paper.

**Supplementary Material**

**Supplementary Figures and Legends:**

**Fig. S1. Perioperative stroke mice exhibited enlarged infarct volumes and increased mortality compared to stroke-only mice after distal middle cerebral artery occlusion.**


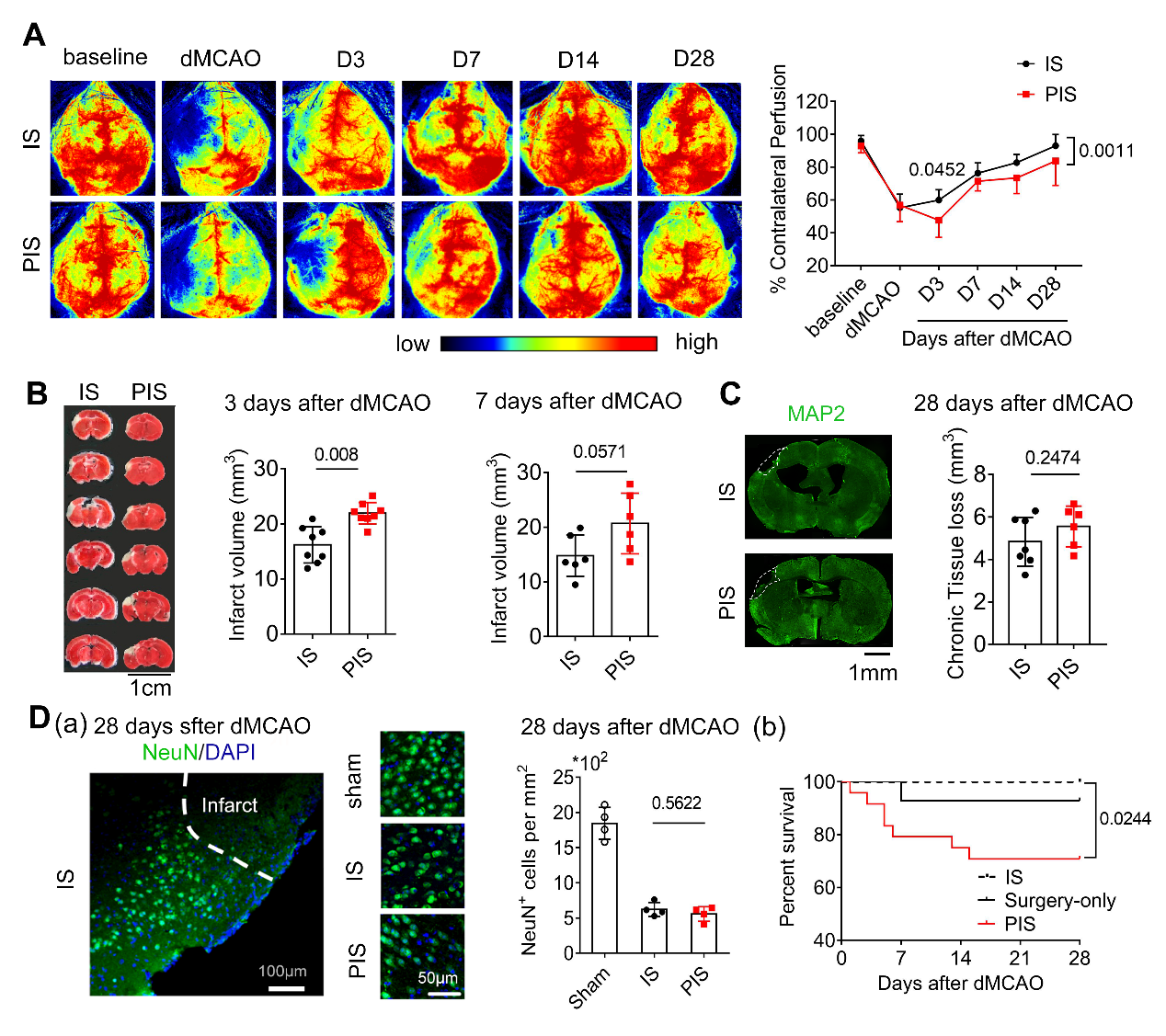


**Fig. S1. Perioperative stroke mice exhibited enlarged infarct volumes and increased mortality compared to stroke-only mice after distal middle cerebral artery occlusion.**

(A) Cerebral blood flow (CBF) was monitored using the 2-dimensional laser speckle technique at 0 d (baseline and after dMCAO), 3 days, 7 days, 14 days, and 28 days after dMCAO. Representative 2-D laser speckle images from the IS and PIS group, respectively. Images represent the axial surface view from the top. The left side of the picture designates the left infarct region. CBF in the infarct region was quantified and expressed as percent change from baseline. n=6-7 per group; repeated measures ANOVA, Bonferroni post hoc correction. (B) Infarct volumes 3 days and 7 days after dMCAO in IS and PIS mice. n=8 per group; unpaired *t* test. (C) Quantification of brain tissue loss at 28 days after dMCAO in PIS and IS mice, as determined by immunostaining for the neuronal marker MAP2. n=6-7 per group; unpaired *t* test. (D) (a) Quantification of NeuN^+^ cells in peri-infarct cortex area of Sham, IS and PIS mice. Left sale bar=100 μm. Right scale bar=50 μm. n=3 per group; one-way ANOVA, Bonferroni post hoc correction. (b) Survival analysis revealed a lower survival rate in PIS mice compared to IS and Surgery-only mice. n=10-15 per group; Kaplan-Meier survival analysis.

**Fig. S2. PIS mice exhibit deteriorated sensorimotor and memory functions after dMCAO.**


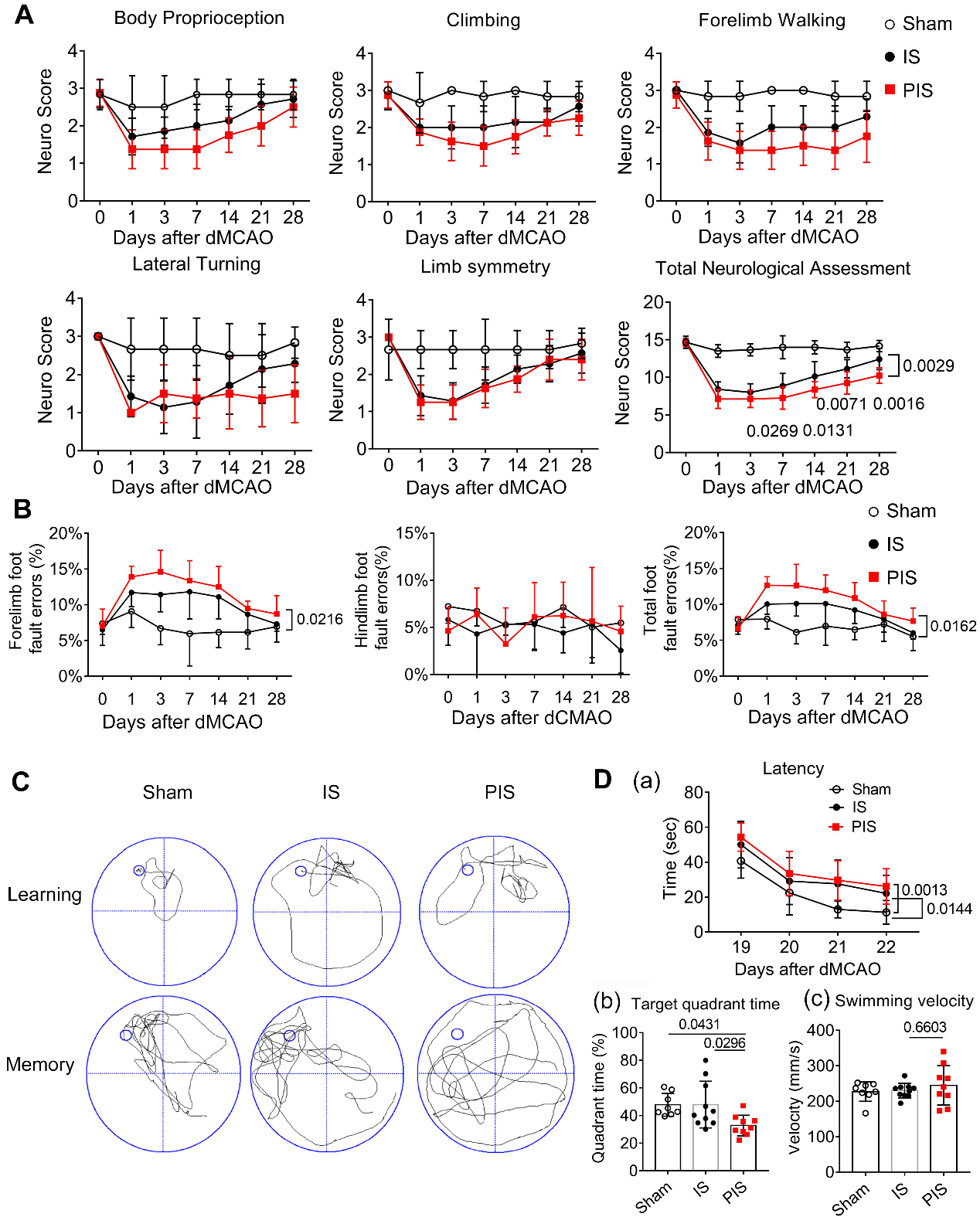


**Fig. S2. PIS mice exhibit deteriorated sensorimotor and memory functions after dMCAO.** (A and B) Sensorimotor functions were evaluated up to 28 days after dMCAO in Sham, PIS and IS mice using the modified Garcia Score (A) and foot fault test (B). n=6-8 per group; repeated measures ANOVA, Bonferroni post hoc correction. (C-D) Long-term cognitive functions were assessed by the Morris water maze. n=8 per group. Representative images of the swim paths of mice in each group while the platform was present (learning phase) and after it was removed (memory phase). Similar learning deficits were observed in PIS and IS mice, as reflected by similar escape times (a). More severe memory deficits were observed in PIS mice, as reflected by shorter time spent in the target quadrant (b). Both groups had similar swim speeds, thereby showing equivalent motor skills (c). One-way ANOVA, Bonferroni post hoc correction.

**Fig. S3. Quantification of different immune cell populations after perioperative stroke.**


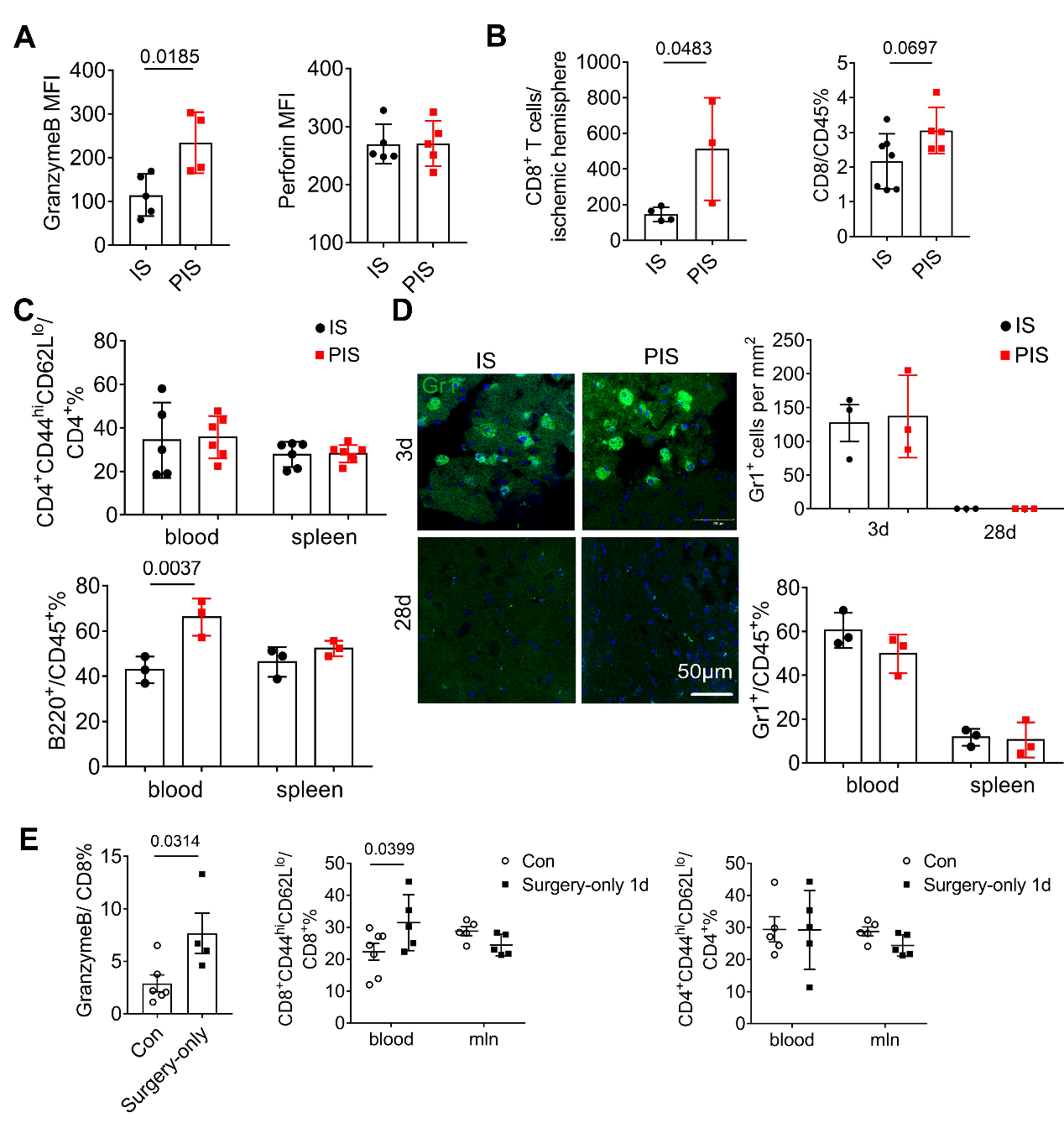


**Fig. S3. Quantification of different immune cell populations after perioperative stroke.** (A) Flow cytometry analysis revealed increased Granzyme B and unaltered Perforin expression in brain-infiltrated CD8^+^ T cells from PIS mice compared to IS mice 7 days after dMCAO. n=4-5 per group. (B) Increased cell number and proportion of brain-infiltrated CD8^+^ T cells from PIS mice compared to IS mice 28 days after dMCAO. n=3-7 per group; unpaired *t* test. (C) The percentages of CD4^+^CD44^hi^CD62L^lo^ were similar in peripheral blood and spleen of PIS mice and IS mice 7 days after dMCAO. n=5-7 per group. The percentage of B220^+^ cells significantly increased in the peripheral blood of PIS mice compared to IS mice. n=3 per group. (D) Representative images and quantification of immunofluorescence staining of Gr1^+^ T cells in PIS and IS mice. Similar numbers of Gr1^+^ cells were detected in both PIS and IS mice. The percentages of Gr1^+^ measured by flow cytometry in both blood and spleen were similar in PIS and IS mice. n=3 per group. (E) The percentage of GranzymeB^+^CD8^+^ T cells and CD8^+^CD44^hi^CD62L^lo^ significantly increased while the CD8^+^CD44^hi^CD62L^lo^ remained unaltered in the peripheral blood of surgery-only mice compared to control mice 1 day after ICR surgery. n=5-7 per group; unpaired *t* test, one-way ANOVA, Bonferroni post hoc correction.

**Fig. S4. Peripheral factors mediated ICR-induced deterioration of ischemic stroke.**


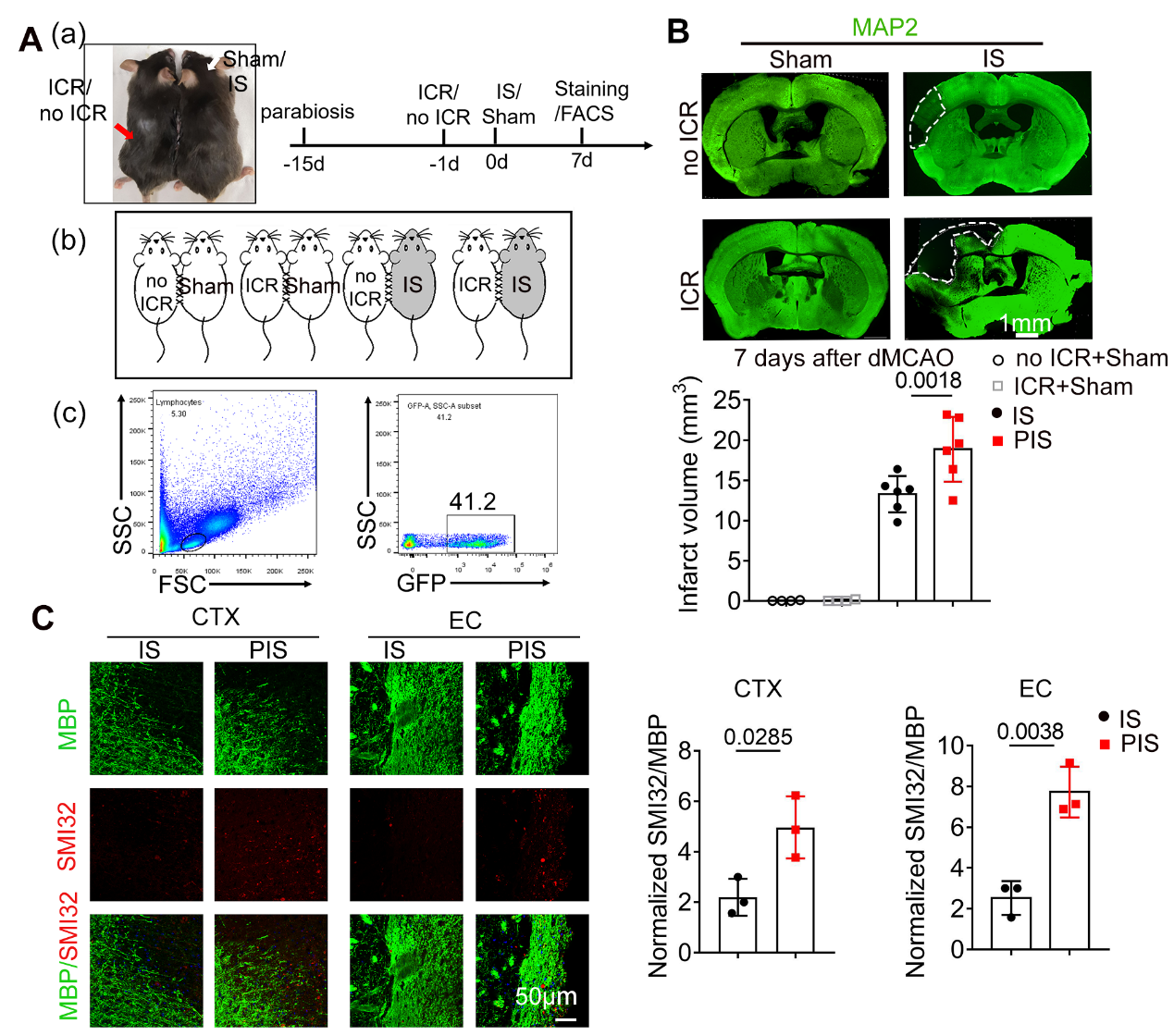


**Fig. S4. Peripheral factors mediated ICR-induced deterioration of ischemic stroke.** (A) Age-matched male C57BL/6J mice were used to randomly generate parabiotic pairs (n=4-6 pairs/group) as illustrated. (a) A representative parabiotic pair and the experimental design. (b) In all pairs, the left mouse was treated with either sham surgery or ICR, while the right mouse received either sham injury or dMCAO. (c) Joining of a WT mouse and GFP positive mouse was used to identify the outcome of the parabiosis method. Flow cytometric analysis of peripheral blood collected from the WT parabiotic 14 days following surgical attachment and approximately half GFP positive cells were revealed in WT mouse. (B) Representative MAP2 staining images at 7 days after dMCAO. The infarct area is indicated by the white dotted line in the left side. Scale bar=1 mm. Quantification of infarct volume. n=4-6 pairs/group. One-way ANOVA, Bonferroni post hoc correction. (C) Representative images of SMI32 (red) and MBP (green) double-immunostaining of cortex and EC area 7 days after dMCAO in IS and PIS pair. Scale bar=50 μm. Upregulated ratio of SMI32/MBP intensities 7 after dMCAO in PIS compared to IS pair. n=3 per group; unpaired *t* test.

**Fig. S5. Similar astrocyte response in PIS and IS mice.**


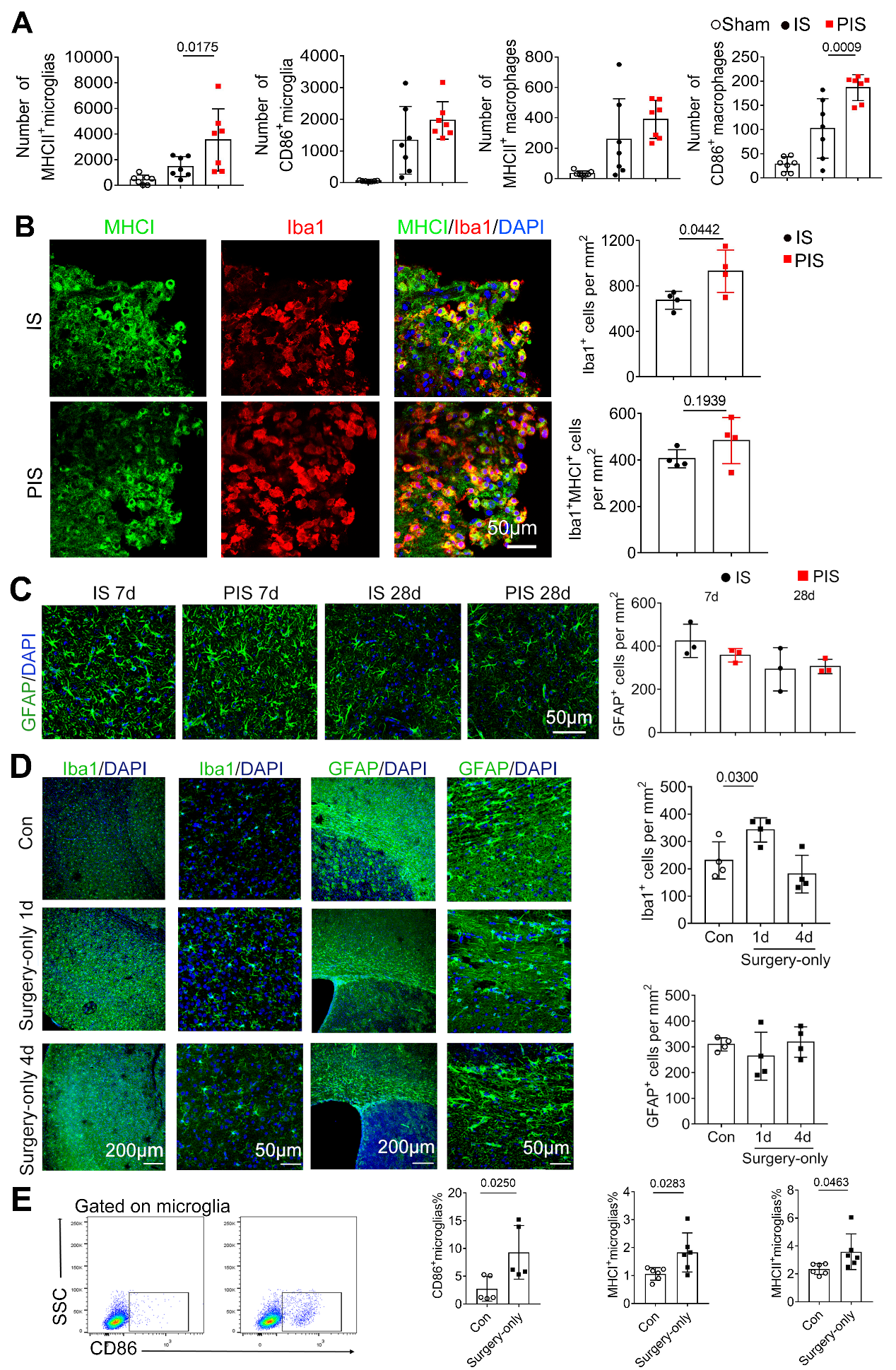


**Fig. S5. Similar astrocyte response in PIS and IS mice.** (A) Quantification of the absolute numbers of MHC II^+^ microglia, CD86^+^ microglia, MHC II^+^ macrophages and CD86^+^ macrophages in sham, IS, and PIS mice. n=7 per group; one-way ANOVA, Bonferroni post hoc correction. (B) Representative images (Left) and quantification (Right) of MHC I (green) and Iba1 (red) staining 7 days after dMCAO in PIS and IS mice. Scale bar=50 μm. n=4 per group; unpaired *t*-test. (C) Representative images (Left) and quantification (Right) of GFAP^+^ positive cells 7 and 28 days after dMCAO in PIS and IS mice. Similar numbers and morphology of GFAP^+^ astrocytes were detected in both groups. Scale bar=50 μm. n=3 per group. (D) Representative images (Left) and quantification (Right) of Iba1^+^ microglia in the cortex and GFAP^+^ astrocytes in CC/EC 1 and 4 days after ICR surgery. Left scale bar=200 μm. Right scale bar=50 μm. n=4 per group; one-way ANOVA, Bonferroni post hoc correction. (E) Quantification of the percentages of CD86^+^ microglia, MHC I^+^ microglia, MHC II^+^ microglia in control and surgery-only mice 1 day after ICR surgery. n=5-6 per group; unpaired *t* test.

**Fig. S6 Uncropped western bolt images for MBP, Olig2, Tublin expression in PIS and IS mice.**


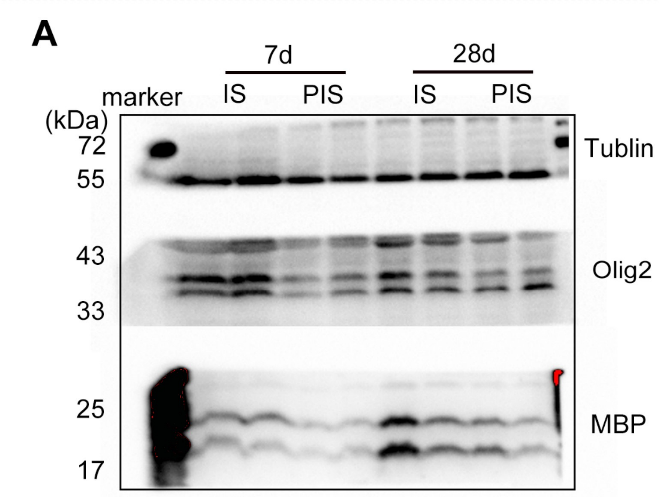


**Fig. S6. Uncropped western bolt images for MBP, Olig2, Tublin expression in PIS and IS mice.** (A) The uncropped western blot images for MBP, Olig2, Tublin 7 and 28 days after dMCAO in PIS and IS mice. Data are summarized in Figure. 1B.

**Fig. S7. Complete blood count and serum Procalcitonin (PCT) after surgery and ischemia.**


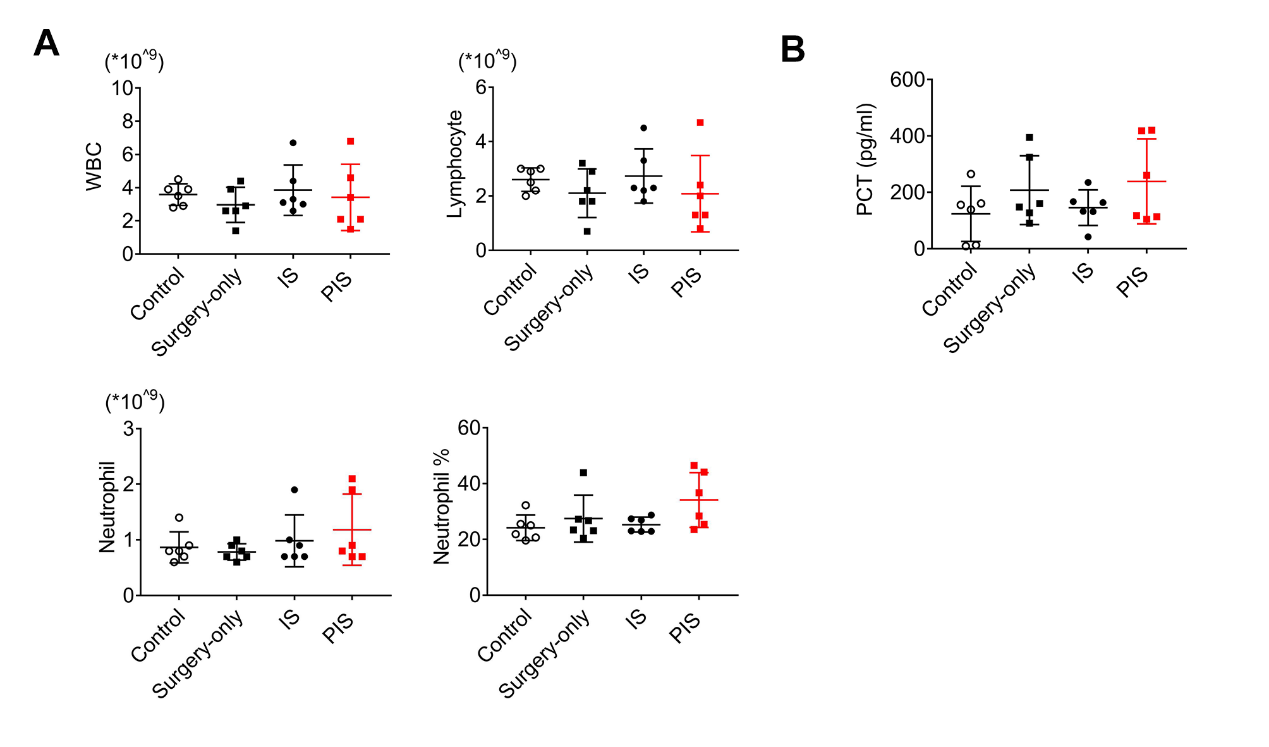


**Fig. S7. Complete blood count and serum Procalcitonin (PCT) after surgery and ischemia.** A-B. Similar cell counts of white blood cell, lymphocyte, neutrophil, percentage of neutrophils (A) and the concentration of PCT (B) in Surgery-only, IS and PIS group, compared with the control mice. n=6 per group, one-way ANOVA, Bonferroni post hoc.

**Fig. S8. Animal weights at baseline and post-operative day 28.**


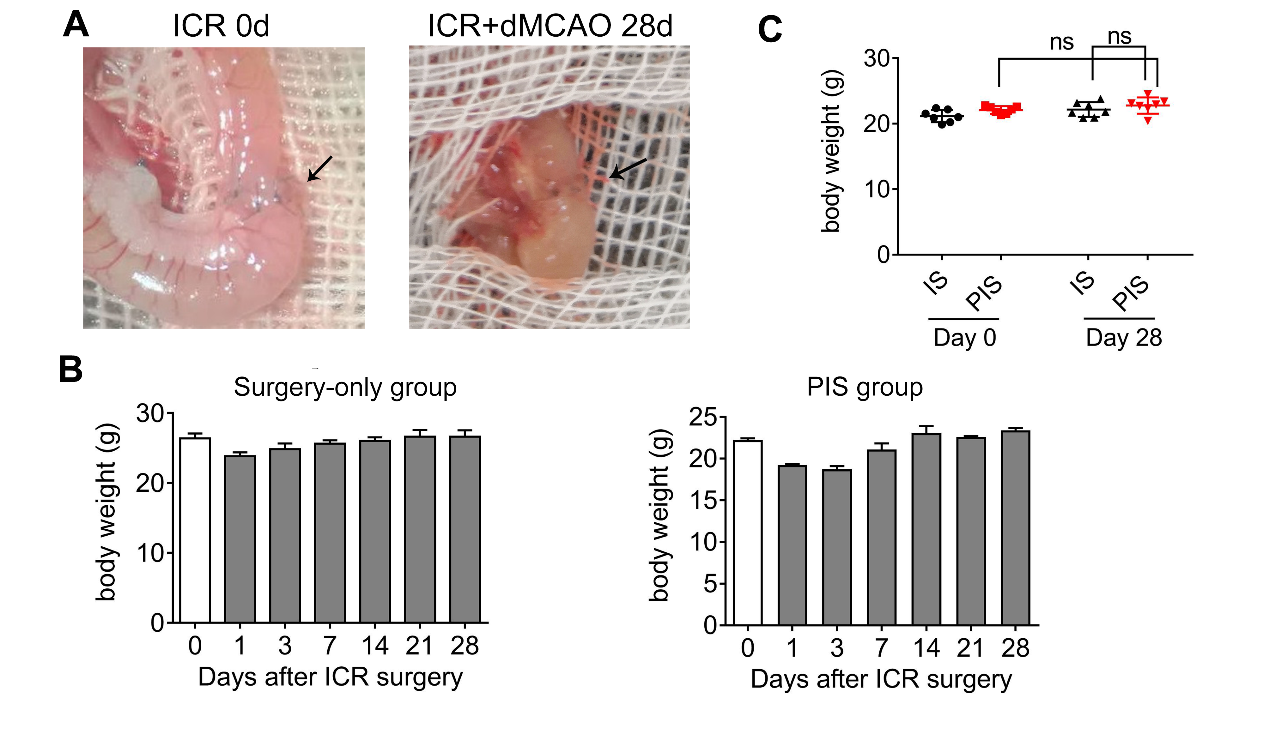


**Fig. S8. Animal weights at baseline and post-operative day 28.** (A) Representative images of completed ileocolic anastomosis immediately and 28 days after surgery. (B) The trends of weights after ICR surgery up to 28 days. n=5-7 mice per group. (C) No significant changes in weights were observed in IS and PIS mice 28 days after dMCAO. n=7 mice per group, two-way ANOVA, Bonferroni post hoc.

**Fig. S9. Identification of the CD8^+^ T cell depletion efficiency.**


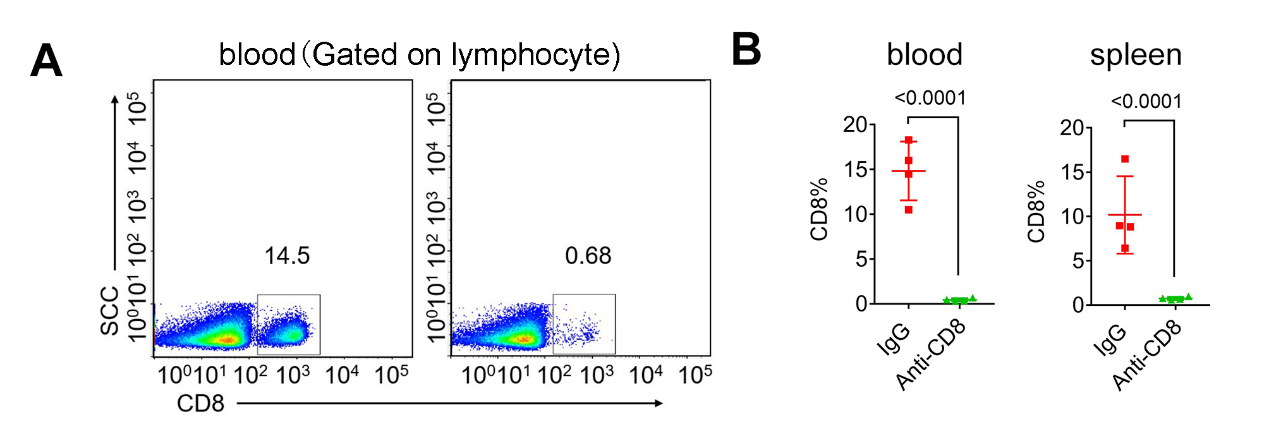


**Fig. S9. Identification of the CD8^+^ T cell depletion efficiency.** A-B. Representative dot plots (A) and quantification (B) of flow cytometry of CD8^+^ T cells in the blood and spleen after CD8^+^ T lymphocytes depletion. n=4 per group; unpaired *t* test.

**Supplementary Table 1. Animal number (survival/total) in each group.**


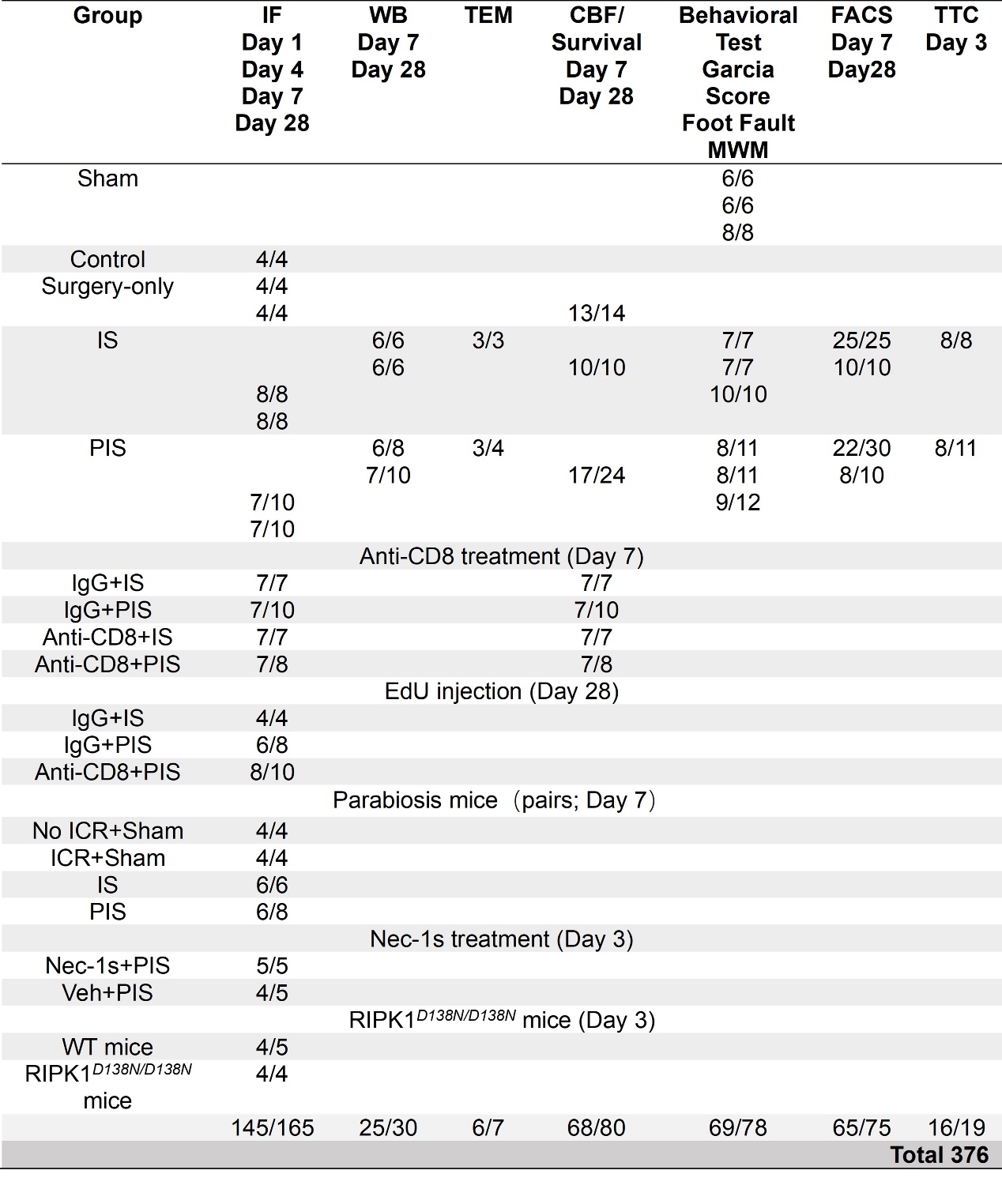
IF: immunofluorescence; WB: Western blot; TEM: Transmission electron microscopy; CBF: cerebral blood flow; MWM: Morris water maze; FACS：flow cytometry; TTC: 2,3,5-Triphenyl tetrazolium chloride staining.

**Supplementary Table 2. Antibodies used for immunofluorescence, Western blot and FACS.**


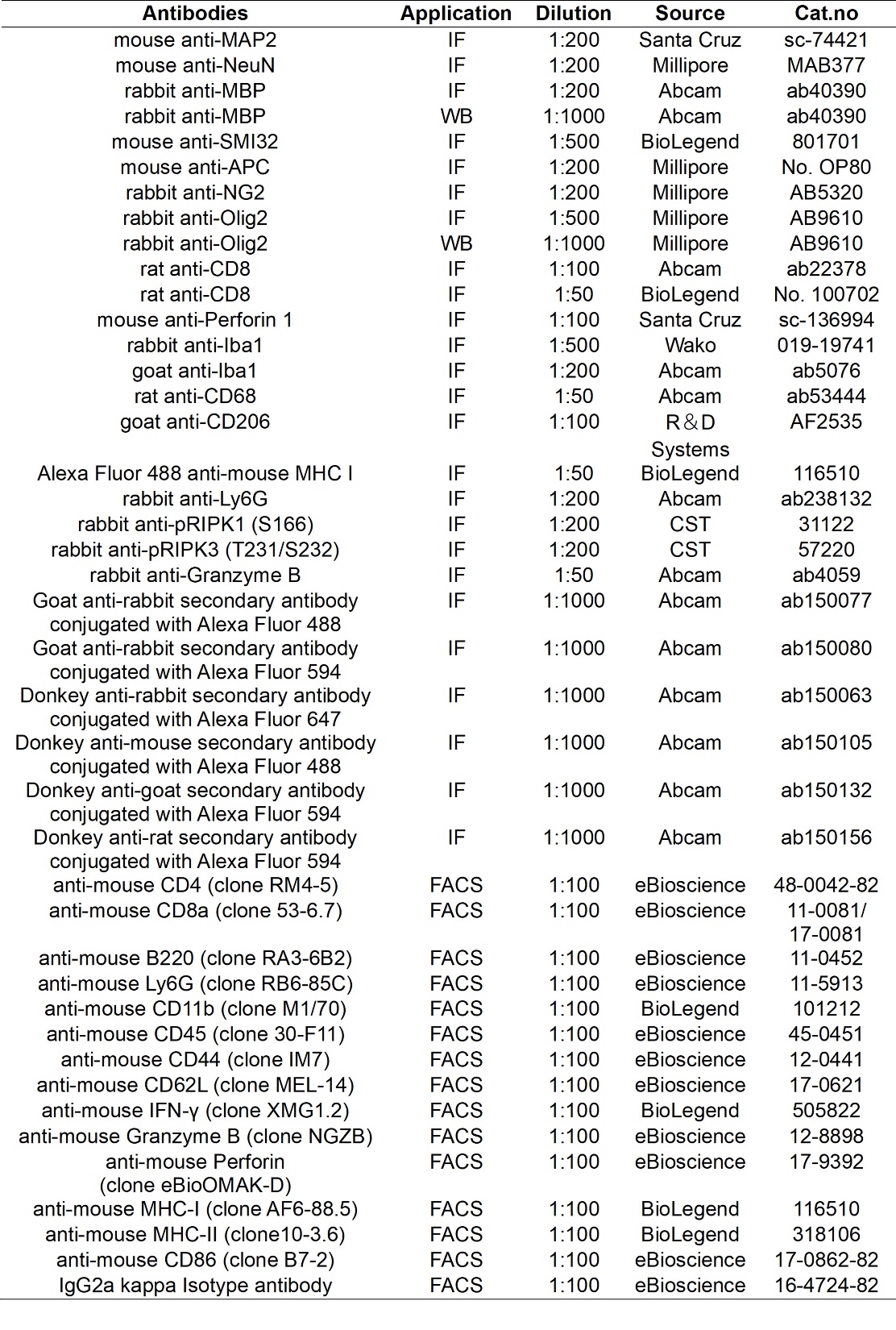


IF: immunofluorescence; WB: Western blot; FACS：flow cytometry.

**Supplementary Methods**

**Transmission electron microscopy (TEM)**

TEM was used to measure myelin thickness in the peri-infarct cortex area. The cortex area near the scar was quickly microdissected into 1 mm^3^ cubes after perfusion with ice-cold PBS from 3 mice in each group 28 days after dMCAO. These cubes were immersion-fixed, dehydrated in increasing concentrations of acetone, and embedded in Araldite resin. Ultrathin 60-nm sections were cut, stained with uranyl acetate and lead citrate, and then viewed using a JEM1400 TEM (JEOL, Akishima, Japan). Three images of each animal were randomly acquired and analyzed with Image J by two observers blinded to experimental groups. At least 50 random axons per mouse were collected to evaluate axon and myelin circumference. The ratio of the inner axonal diameter to the total outer diameter (axonal diameter + total myelin sheath thickness) was calculated as G-ratios.

**EdU injections**

Adult male mice were randomly distributed to IgG+IS, IgG+PIS and Anti-CD8+PIS groups before dMCAO. To label proliferating cells, the thymidine analogue EdU (5-ethynayl-2’-deoxyuridine, 50mg/kg, BCK594-IV-IM-S, baseclick, Germany) was intraperitoneally injected once daily in 7 days,14 days and 21 days after ischemia induction in all above groups.

**Flow cytometry**

Mice were first euthanized using sodium pentobarbital (100mg/kg) and then perfused with cold PBS. Brains without meninges were dissociated and the ischemic ipsilateral were collected 7 or 28 days after dMCAO. Brain homogenates were prepared with the Neural Tissue Dissociation Kit (T) using a gentle MACS dissociator (Miltenyi Biotec) following the manufacturer’s instructions. The suspension was applied to a 70μm MACS SmartStrainer and resuspended in 30% Percoll. Cell suspensions in 30% Percoll were overlaid on the 70% Percoll gradient followed by gradient centrifugation. After collecting the cells at the interface, single cells were incubated with antibodies to surface antigens, and then permeabilized/fixed using an intracellular staining kit (eBioscience, 88-8824). Isolation of immune cells from peripheral blood, spleens and mesenteric lymph nodes (MLN) was performed 7 and 28 days after dMCAO. Spleens and mesenteric lymph nodes were mechanically dissociated for single cell suspensions and passed through a 70μm filter after RBC lysis buffer (eBioscience). For effective cytokine induction, blood, mesenteric lymph nodes, or brain cells were stimulated with cell stimulation cocktail (Invitrogen, 00-4975) for 6 hours and then stained with respective antibodies (IFN-γ, Granzyme B, and Perforin). FACS analysis was performed on a BD FACSVerse and the data was analyzed using FlowJo (V10). Gates were set according to unstained samples and isotype controls.

**Immunofluorescence staining, image acquisition and analysis**

The floating coronal brain tissue sections were permeabilized with 1% Triton X-100 in PBS (PBST) and then blocked using 0.3% Triton X-100 in PBS with 5% donkey serum for 1 hour at room temperature. Sections were further incubated with an unconjugated affinity purified F(ab) fragment anti-mouse IgG (H+L) (1:100, Abcam, ab6668) for 1 hour at room temperature to reduce background when the mouse primary antibodies were used. Primary antibodies were incubated overnight at 4℃, followed by three washes in 0.3% PBST. The sections were then incubated with the appropriate secondary antibodies (1:1000) for 6 hours at 4℃. Finally, the sections were washed and mounted with DAPI Fluoromount-G^®^ (Southern Biotech, 0100-20). We performed secondary antibody-only control staining to exclude non-specific binding.

To quantify the numbers of GFAP, Iba1, APC, NG2 and Olig2 positive cells, three randomly selected microscopic fields in the peri-infarct areas of the cortex were assessed for each mouse. To quantify MBP and SMI32 intensity, two randomly selected microscopic fields in the peri-infarct areas of cortex, two in the infarct areas of external capsule (EC) and striatum were assessed for each mouse. MAP2 staining of six consecutive sections per mouse 28 days after dMCAO was captured to measure the chronic neuronal tissue loss.^1^ To determine the proximity of Iba1^+^ cells to CD8^+^ T cells, Iba1^+^ cells were selected manually, from which a circle within a 20μm radius was drawn. We then quantified the numbers of CD8^+^ T cells located within that circle. The peri-infarct area was identified as the tissue that involves a radial distance of 200-300μm from the margin of the infarct, as previously described.^2^ Images from three to five mice were gathered for quantitative analysis and two observers blinded to the grouping manually quantified the images by Image J (1.52a).

**Primary antibodies and reagents**

The following antibodies were applied: mouse anti-MAP2 (1:200, Santa Cruz, sc-74421), mouse anti-NeuN (1:200, Millipore, MAB377), rabbit anti-MBP (1:200, Abcam, ab40390), mouse anti-SMI32 (1:500, BioLegend, 801701), mouse anti-APC (1:200, Millipore, No. OP80), rabbit anti-NG2 (1:200, Millipore, AB5320), rabbit anti-Olig2 (1:500, Millipore, AB9610), rat anti-CD8 (1:100, Abcam, ab22378), rat anti-CD8 (1:50, BioLegend, No. 100702), rabbit anti-Iba1 (1:500, Wako, 019-19741), goat anti-Iba1 (1:200, Abcam, ab5076), mouse anti-Perforin 1 (1:100, Santa Cruz, sc-136994), Alexa Fluor 488 anti-mouse MHC I (H-2Kb, 1:50, BioLegend, 116510), rabbit anti-Ly6G (Gr1, 1:200, Abcam, ab238132), rabbit anti-Granzyme B (1:50, Abcam, ab4059), rat anti-CD68 (1:50, Abcam, ab53444), goat anti-CD206 (1:100, R＆D Systems, AF2535), rabbit anti-pRIPK1 (S166, 1:200, CST, 31122), rabbit anti-pRIPK3 (T231/S232, 1:200, CST, 57220), anti-mouse IgG (H+L) (1:100, Abcam, ab6668), anti-rabbit secondary antibody conjugated with Alexa Fluor 488 (1:1000, Abcam, ab150077), anti-rabbit secondary antibody conjugated with Alexa Fluor 594 (1:1000, Abcam, ab150080), anti-rabbit secondary antibody conjugated with Alexa Fluor 647 (1:1000, Abcam, ab150063), anti-mouse secondary antibody conjugated with Alexa Fluor 594 (1:1000, Abcam, ab150108), anti-mouse secondary antibody conjugated with Alexa Fluor 488 (1:1000, Abcam, ab150105), anti-goat secondary antibody conjugated with Alexa Fluor 594 (1:1000, Abcam, ab150132), anti-rat secondary antibody conjugated with Alexa Fluor 594 (1:1000, Abcam, ab150156).

For flow cytometric analysis, single-cell suspensions were stained with the following antibodies: anti-mouse CD4 (clone RM4-5, eBioscience), CD8a (clone 53-6.7, eBioscience), B220 (clone RA3-6B2, eBioscience), Ly6G (clone RB6-8C5, eBioscience), CD11b (clone M1/70, BioLegend), CD45 (clone 30-F11, eBioscience), CD44 (clone IM7, eBioscience), CD62L (clone MEL-14, eBioscience), IFN-γ (clone XMG1.2, BioLegend), Granzyme B (clone NGZB, eBioscience), Perforin (clone eBioOMAK-D, eBioscience), MHC-I (clone AF6-88.5, BioLegend), MHC-II (clone 10-3.6, BioLegend), CD86 (clone B7-2, eBioscience), and the appropriate isotype control, following the manufacturer's protocols.

The following reagents were also used in this study: CD8α-specific monoclonal antibody (clone 53-6.72, BioXCell), EdU in vivo Kits (baseclick, BCK594-IV-IM-S), Cell Stimulation Cocktail (Invitrogen, 00-4975), DAPI Fluoromount-G^®^ (Southern Biotech, 0100-20), TEM fixing buffer (Servicebio, G1102), O.C.T compound (Sakura, 4583).

**References**

1. Ma S, Wang J, Wang Y, Dai X, Xu F, Gao X, et al. Diabetes mellitus impairs white matter repair and long-term functional deficits after cerebral ischemia. *Stroke*. 2018;49:2453-2463

2. Zhang Q, Zhu W, Xu F, Dai X, Shi L, Cai W, et al. The interleukin-4/ppargamma signaling axis promotes oligodendrocyte differentiation and remyelination after brain injury. *PLoS Biol*. 2019;17:e3000330
